# Improved Assessment of Globularity of Protein Structures and the Ellipsoid Profile of the Biological Assemblies from the PDB

**DOI:** 10.3390/biom13020385

**Published:** 2023-02-17

**Authors:** Mateusz Banach

**Affiliations:** Department of Bioinformatics and Telemedicine, Faculty of Medicine, Jagiellonian University Medical College, Medyczna 7, 30-688 Kraków, Poland; mateusz.banach@uj.edu.pl

**Keywords:** bioinformatics, biological assembly, bounding ellipsoid, globularity, kernel density, nearest neighbor search, protein complex, protein domain, principal component analysis

## Abstract

In this paper, we present an update to the ellipsoid profile algorithm (EP), a simple technique for the measurement of the globularity of protein structures without the calculation of molecular surfaces. The globularity property is understood in this context as the ability of the molecule to fill a minimum volume enclosing ellipsoid (MVEE) that approximates its assumed globular shape. The more of the interior of this ellipsoid is occupied by the atoms of the protein, the better are its globularity metrics. These metrics are derived from the comparison of the volume of the voxelized representation of the atoms and the volume of all voxels that can fit inside that ellipsoid (a uniform unit Å cube lattice). The so-called ellipsoid profile shows how the globularity changes with the distance from the center. Two of its values, the so-called ellipsoid indexes, are used to classify the structure as globular, semi-globular or non-globular. Here, we enhance the workflow of the EP algorithm via an improved outlier detection subroutine based on principal component analysis. It is capable of robust distinguishing between the dense parts of the molecules and, for example, disordered chain fragments fully exposed to the solvent. The PCA-based method replaces the current approach based on kernel density estimation. The improved EP algorithm was tested on 2124 representatives of domain superfamilies from SCOP 2.08. The second part of this work is dedicated to the survey of globularity of 3594 representatives of biological assemblies from molecules currently deposited in the PDB and analyzed by the 3DComplex database (monomers and complexes up to 60 chains).

## 1. Introduction

Molecular simulations are crucial in silico tools for the investigation of the structure and function of biological systems. Protein molecules perform critical roles in those systems. Problems with their activity, for instance caused by misfolding or abnormal aggregation, can lead to detrimental or fatal states of a person’s health [1]. These ongoing challenges stimulate scientists to look for new drugs, but also for early detection and prediction models. One of the biggest recent advances is AlphaFold [2,3], a robust and accurate machine learning package for protein structure prediction. AlphaFold2 outperformed its competition in 2020 during the 14th iteration of the CASP initiative [4].

Molecular mechanics (MM) is a computer simulation technique in which atoms of a protein (or another molecule) are represented by rigid balls connected by springs, which represent the bonds [1,5]. The state of the system can be then described through the potential energy function of atomic coordinates. The commonly used MM force fields invoke the potential energy equation as a sum of bonded and non-bonded terms. Each term has its own functional form. The non-bonded potentials (e.g., electrostatic, van der Waals and hydrogen bond interactions) are typically calculated on the basis of pairs of atoms unless some form of coarse-grained approximation is employed [6,7]. These potentials express that part of the total potential energy that is not already covered by the bonded terms (e.g., bond stretching, bending and torsional rotations). Together with a set of parameters and other factors specific to the force field, they localize the current state of the system—also known as its snapshot [5]—on the folding landscape [8]. This mapping acts as a guide for the atoms of the molecule, allowing them to move toward the (still reachable) conformations corresponding to the local energy minima. It is a method of optimization that finds its use in structural refinement and relaxation, but also in the prediction of the native tertiary (i.e., folding) and quaternary (i.e., docking) structure of proteins [9].

In molecular dynamics (MD), Newton’s equations of motion are integrated to allow the static MM ball and spring models to fluctuate [1], producing a trajectory (i.e., a history of changes of the atomic coordinates during the specified time period) that gives an insight into the kinetics and thermodynamics of the simulated system [5].

One of the properties to consider during the computer modeling of proteins is their relationship with the surrounding aqueous environment that affects them (and vice versa) [10,11,12,13]. Hence, to accurately address the biological problems involving those molecules, one must be capable to somehow represent the solvent in the simulation [14]. In other words, a water model is needed. There are two types of such models: explicit and implicit. Explicit solvation puts the protein in a box filled with discrete H_2_O molecules. It provides the highest allowed accuracy at the expense of highest computation time (i.e., the computer can spend more time on the motion of water than on the motion of the protein [14]). Conversely, in the implicit solvation the aqueous environment is defined as a uniform, infinite, high-dielectric medium that surrounds the low-dielectric solute. Implicit models can be orders of magnitude faster to compute (i.e., they have no H_2_O molecules to track), but as stronger approximations, they come at the cost of a reduced accuracy with respect to the explicit water representation [14]. Nevertheless, the advantages of implicit solvation have brought many models [15]. Solvent effects are embedded in the popular approaches via solvation free energy of the protein, in which the non-polar contribution (i.e., the perturbation in the continuous structure of water) is a function of SASA, the solvent-accessible surface area [1,14,15]. The practicality of this method comes from the fact that it can be readily incorporated in the force field energy equations.

The solvation free energy of a realistic biomolecule is “(…) some complex function of molecular conformation (…)” [14]. It can be understood as the need to look at the protein–water relationship also from the perspective of the whole tertiary/quaternary structure (or its relevant parts, e.g., domains) to obtain another view of the specificity of the associated biological activities [16]. Fuzzy oil drop (FOD) [17,18,19], our in-house model of the density of hydrophobicity, tries to fulfill this purpose. It differs from the SASA-based models in its approximation of the shape of the proteins with an ellipsoid (also known as the drop) that separates the solute from the implicit solvent. The size of this ellipsoid is used to gauge the expected hydrophobicity of the residues via the 3D Gaussian function. It is highest in the center and decrements toward zero near and beyond the surface. This “theoretical” distribution of hydrophobicity (*T*) is put against its “observed” counterpart (*O*), which is derived from the residue–residue hydrophobic interactions. The strength of those interactions is modeled via Michael Levitt’s sigmoid function [20]. Comparison of the *T* and *O* distributions can uncover residues responsible for hydrophobic core stability or instability, for instance due to their involvement in protein–protein or protein–ligand interactions [21]. On the other hand, the metrics of the FOD model do not translate to atomistic energy terms and cannot be directly introduced into the common force fields.

This paper is focused on the characteristic feature of the FOD model—the ellipsoid. Or, more precisely, the ellipsoidal approximation of the shape of proteins. The theory of the FOD model assumes that its input structure is globular and has folded in accordance with the hydrophobic effect—that its hydrophobic residues are in the core, isolated from the solvent by a sheath of their polar counterparts. Proteins that do not conform to this scheme, such as those with non-globular conformations or with too much exposition of hydrophobicity on their surface, are pronounced by the FOD model as devoid of a stable hydrophobic core. When measuring the magnitude of this accordance/discordance, it is useful to know if the input structure is actually globular. It is also possible that some of its fragments may cause issues with the ellipsoidal representation, skewing its metrics of hydrophobicity. Knowing this is particularly crucial during mass scale calculation when the manual inspection of hundreds or thousands of proteins is impractical. However, because the FOD model does not natively track the globularity property, a different tool is needed, like another in-house algorithm—the ellipsoid profile [22].

Ellipsoid profile (EP) is a simple method for the quantitative measurement of the globularity of proteins. The globularity property is understood in its context as the ability of the input structure to occupy a minimum volume enclosing ellipsoid (MVEE) [23] fit to it. The EP algorithm converts atoms of the protein into disjointed voxels (i.e., non-intersecting 1 Å^3^ cubes) and compares them with all disjointed voxels that can fill that MVEE (also known as the grid—a uniform 1 Å^3^ cube lattice). Each voxel is weighted by its corresponding value of the ellipsoid equation to account for the uneven shape of the molecular surface without actually calculating that surface (just like it is not calculated in the FOD model). The structure can be then classified as globular, semi-globular or non-globular. This workflow is readily applicable to any kind of protein and even non-protein input.

The baseline EP algorithm [22] is recalled in Section 2.3. The binding of the protein in an MVEE and the calculation of the globularity metrics (ellipsoid indexes and profiles) is preceded by an optional outlier detection subroutine. When enabled, it looks for residues that protrude from the main body of the protein, for example in the form of disordered chain fragments fully exposed to the solvent. Only the non-outlier residues (also known as the guides) are then passed to the MVEE algorithm. The current method of their selection is based on kernel density estimation (KDE) [24,25]. The detection subroutine additionally forestalls some of the local features of the molecular surface of the protein (e.g., exposed side chains) from negatively affecting the ellipsoid bounding, resulting in a tighter, more befitting representation that is closer to what a human would use to describe the overall globularity of the analyzed structure on the basis of its visual inspection.

We noticed, however, that the KDE-based approach can sometimes run into trouble with the detection and proper isolation of outlier residues that gather in small clusters. We have recently [26] introduced to the FOD algorithm an alternative method of bounding the protein in the drop that proficiently handles the exposed, low-density regions of the structure, making this process less sensitive to their presence. It utilizes principal component analysis (PCA) [27] in tandem with outlier detection based on confidence ellipsoid [28]. Since it works well for FOD, we decided to try it in the context of the analysis of structural globularity too. A new, enhanced with PCA version of the EP algorithm is described in Section 3.1 and tested on six example proteins in Section 3.2.

The baseline EP algorithm was previously [22] applied to the representatives of 2124 protein domain superfamilies from a modified ASTRAL compendium [29,30] of the SCOP database [31,32] (Section 2.1). The performance of the modified EP algorithm from Section 3.1 was tested on the same structural dataset using the original, KDE-based output as a reference. The results are presented and discussed in Section 3.3.

The second part of this contribution is dedicated to the initial survey of the landscape of the EP-based globularity of the protein biomolecules. These biomolecules were understood here as the manually assigned (i.e., not automatically matched by software) biological assemblies from structures currently deposited in the Protein Data Bank (PDB) [33,34]. Just like domains are structurally independent parts of proteins with their own hydrophobic cores [35] (although that is only one their defining concepts [36]), these assemblies correspond to the functionally relevant forms of those proteins [37,38]. Thus, they are of special scientific interest and constitute a well-founded input for the EP algorithm. Their analysis complements the results for the ASTRAL domain representative subset.

The instructions for how to extract a non-redundant set of biomolecules from the PDB in terms of the structure (i.e., the property that matters to the EP algorithm) is given in the first part of Section 3.4. We based their selection on the data from the 3DComplex 6.0 database [39,40], domain fingerprints at the SCOP superfamily level and the quality of the model (i.e., resolution and the number of errors in the crystallographic experiment). One can think of this collection as an analogue to the ASTRAL compendium only from the perspective of the quaternary structure. Monomers and complexes up to 60 chains were included in it, resulting in 3594 representatives of a total of 136,608 biological assemblies. The analysis of their globularity is conducted in the second part of Section 3.4.

## 2. Materials and Methods

### 2.1. The Protein Database

A total of 5481 PDB files downloaded from the Protein Data Bank (PDB) [33,34] were processed during this research. They were split into two groups of 1901 and 3577 structures, respectively. The total number of inputs for the EP algorithm was 5718.

#### 2.1.1. The 2124 Representatives of Domain Superfamilies

The group of 1901 PDB structures contained the representatives of 2124 SCOP domain superfamilies used in the testing of the improved version of the EP algorithm. This collection was previously [22] compiled as a small modification of the domain superfamily representative subset from the ASTRAL compendium [29,30] released along SCOP 2.08 [31,32] (2.08 was the latest stable version of SCOP as of December 2022). The complete changelog can be found in ref. [22]. In short, we omitted the 7 multi-chain genetic domains, replaced 43 structures marked as obsolete by the PDB and added 66 representatives of the coiled coil domain superfamilies (i.e., the members of SCOP class *h*). No other PDB entry from this collection was marked as obsolete since last year.

It should be mentioned here that, by accident, the genetic domain d1aik.1 [41] from SCOP family h.3.2.1 was previously [22] included in the database as the representative of the virus ectodomain superfamily. In hindsight, it should have been replaced with another h.3.2.1 domain only with both chains annotated as one (n.b., each consisting of a single helix), but because it had no negative impact on a global scale, it was kept to facilitate a simple comparison with the former results (i.e., 1:1 in terms of PDB codes). These former results can be found in the Supplementary Materials of ref. [22].

The average crystallographic resolution in this set was 1.75 Å (*σ* = 0.62 Å). A total of 159 structures were obtained via solution NMR. In their case, only the first conformer in the ensemble was used in this study (it also applied to the proteins from Section 2.1.3).

#### 2.1.2. The 3594 Representatives of Biological Assemblies

The group of 3577 PDB structures contained the 3594 manually assigned biological assemblies used for the expansion of the EP-based survey of the globularity from domains to entire proteins. These assemblies represent (or are believed to represent) the functionally relevant forms of proteins that can be encountered in vivo. Monomers and complexes up to 60 chains comprised this set. Redundancy was avoided through the application of two criteria. The first criterion allowed only one biological assembly with a given domain fingerprint at the SCOP superfamily level. These custom fingerprints were compiled from SCOP 2.08 updated on the 21 September 2022. The second criterion allowed only one representative of each quaternary structure (QS) family cluster in the 3DComplex 6.0 database [39] (6.0 was the latest stable version of 3DComplex as of December 2022). Assemblies with complex symmetry assigned by the PDB determined or suspected by 3DComplex maintainers to be incorrect were discarded. Cluster information was extracted from file NRX_0_5_topo_label_clusters.txt (i.e., the QS family clusters in the bottom-up hierarchy) downloaded from the 3DComplex website [40].

The average crystallographic resolution in this set was 2 Å (*σ* = 0.56 Å). All structures were obtained via X-ray diffraction since 3DComplex does not process NMR models.

#### 2.1.3. The 6 Example Proteins

Lastly, some example proteins from refs. [22,26] made another appearance. They are included in the modified domain superfamily representative subset (Section 2.1.1) and were again used to illustrate various combinations of the input and output of the EP algorithm, primarily in Section 3.1 and Section 3.2. Their basic information is given in Table 1 and their 3D renders are shown in Supplemental File S1—Figures S1–S6, while their relevant features are described below.

- Endoglucanase A (PDB code: 1IS9), is a round, relatively large monomer. Use of the outlier residue detection methods should minimally affect its globularity metrics.
- Uncharacterized Protein (PDB code: 3BPD) is a toroidal homoheptamer. It can be easily put inside a bounding ellipsoid but also has a large cavity in its middle. The presence of this cavity should be decipherable from the globularity metrics.
- dUTPase YncF (PDB code: 4B0H) is a homotrimer with long, disordered C-terminal regions at M119–K144. They protrude from the monomers, but in the complex they wrap around the neighbor chains to secure the entire globular motif.
- HLA-DR Invariant Chain (PDB code: 1IIE) is another homotrimer with disordered fragments of the chains (S181–K192 regions at the C-termini) stretching away from the highly globular center of the mass of the molecule. This central “ball” is dense only in the complex—the monomers have a relatively loose tertiary structure.
- Alpha Synuclein (PDB code: 2KKW) is the biologically active (micelle-bound) form of Alpha Synuclein. It is a non-globular chain with a very loose tertiary structure. Its disordered C-terminal region at G101–A140 freely bends out of the plane formed by the two alpha helices that constitute the main body of the molecule.
- Ribosomal Protein L9 (PDB code: 1DIV) is the only multi-domain protein (M1–Q55 and R56–K149) in this group. Its homodimer, shaped like the letter Y, is non-globular.

### 2.2. The Structure Reference Terminology

For brevity, from now on, the structures from the PDB are referred to in this paper by their PDB codes rather than by their names, e.g., 1IS9 instead of Endoglucanase A.

If only PDB code is mentioned, it refers to the entire content of this PDB file, for example, to the monomer of 2KKW, to the trimer of 1IIE and to all 14 chains in 3BPD. A specific chain is pointed to by the *XXXX:Y* notation, where *XXXX* is the PDB code and *Y* is the chain identifier. Thus, 4B0H:B means chain B from 4B0H. The two alternative notations for multiple chains are *XXXX:(Y+Z)* and *XXXX:(Y–Z)*. 1DIV:(A+B) and 1DIV:(A–B) are therefore the explicit labels for the 1DIV dimer and 3BPD:(A–G) denotes the 7 chains that constitute the first of the two identical biological assemblies in 3BPD.

The term “complex” should be understood in the context of this work as a reference to a portion or the entirety of the quaternary structure of given protein that is connected by inter-chain non-bonded contacts. 1DIV is a complex of two chains. It is possible that non-protein molecules (ligands, etc.) were involved in those complexes, but their presence was ignored during this experiment. Likewise, no protein–nucleic complexes were included in the final forms of the input databases described in Section 2.1.

### 2.3. The Ellipsoid Profile Algorithm

This is a description of the initial version of the ellipsoid profile (EP) algorithm [22]. It is presented here to further establish its main concepts, share additional explanations and add a minor enhancement—the new labels for the classes of globularity. The various subroutines that appear in the computational workflow of the EP algorithm (MVEE, convex hull, kernel density estimation and nearest neighbor search) are more thoroughly described in ref. [22]. It is assumed here that the reader is familiar with them. Apart from MVEE, none of them require special tuning for the protein content.

#### 2.3.1. Preparation of the Structure

The EP algorithm requires a set of atomic coordinates and their vdW radii to operate, just like the methods that determine the solvent-accessible surface areas. These coordinates typically originate from PDB structure files. As such, they first need to be freed from artifacts. The application should remove all H_2_O molecules and alternative atom locations other than the most often occupied (n.b., in case of identical values of occupancy, the rotamer with lowest ID, usually A, can be selected). The MODRES records from the PDB header should be then parsed to differentiate the ligands and the modified polymer residues and to match those residues with their standard parents, e.g., MSE with MET.

In the next step the user selects the actual input of the algorithm—the interesting part of the loaded protein structure. It can be any collection of residues (including non-protein), but it is usually a domain, chain or complex. Missing atoms are permitted.

Lastly, each selected residue is paired with its effective atom. An effective atom is a pseudo atom located at the average position of all atoms except hydrogen.

#### 2.3.2. Minimum Volume Enclosing Ellipsoid

The effective atoms of the selected part of the molecule are subsequently bound in a minimum volume enclosing ellipsoid (MVEE). They are used instead of the actual atoms of the protein for a specific reason. As a coarse-grained representation of the residues, they are slightly shifted toward the backbone (i.e., away from the end of the sidechains). Passing them to the MVEE algorithm results in compacter fittings that are less afflicted by the local features of the molecular surface. Put differently, the effective atoms allow the generation of ellipsoids that are—in our opinion—closer to those parts of the structure which a human would use to describe its globularity. Because the MVEE algorithm treats all its input points equally, when the actual atoms are used, those small outliers (such as the protruding sidechains) increase the size of the output ellipsoid, making it larger and emptier than necessary instead of tightly enveloping the molecule. The ellipsoid radii length difference (effective atoms vs. actual atoms) can reach up to 3 Å. It is a consequence of the application of the convex hull algorithm (see next paragraph), which is needed as MVEE is a relatively CPU-intensive procedure. We believe that sacrificing some of the external detail is a better choice than the misrepresentation of the entire molecule. However, it does not mean that a lot of the actual atoms of the protein are excluded from the bounding ellipsoid and do not participate in the subsequent measurement of globularity. In fact, there are usually only a few of them left outside (with respect to the total number of input atoms), giving yet another argument in favor of the use of effective atoms.

We employ two methods of reduction of the calculation time of MVEE. First is the aforementioned passing of the convex hull of the effective atoms as its input. The second is the use of *ε* = 0.01. *ε* is the convergence cutoff parameter for the (1 + *ε*)-approximation of the true MVEE that is actually calculated here. The convex hull saves a lot of time (by orders of magnitude) at a negligible loss of accuracy. *ε* = 0.01 balances well in time and space for the EP algorithm (so to speak). In comparison with the calculation at *ε* = 0.001, the radii of the output ellipsoid can differ by ≈0.5 Å, but are obtained faster by another order of magnitude (e.g., in 0.005 s instead of 0.05 s on an average laptop computer) with only a tiny difference in the globularity metrics. We see it as a worthy trade-off.

#### 2.3.3. Detection of Outlier Residues

Before the effective atoms are encased in the MVEE, an additional step may be performed at user’s discretion—the detection of structural outliers. This subroutine discovers residues that are strongly protruding from the dense, “main” body of the molecule. The disordered chain termini of 1IIE are the prime example of this phenomenon. If not omitted from the MVEE input, these “global outliers” can significantly increase the size of the output ellipsoid (more than the aforementioned “local outliers”), resulting in even more skewed metrics of globularity. In the EP algorithm, a protein shaped like a “ball with a tail” is considered globular, just only in the “ball” part. Thus, this subroutine checks for the presence of such a “tail” and allows the user to keep it or skip it.

The current approach to the outlier finding is based on KDE (kernel density estimation) with a Gaussian kernel and automated bandwidth selection. The less neighbors an effective atom has in its spatial vicinity, the lower is the kernel density assigned to its position in space. Effective atoms with density below a certain threshold are assumed to be outliers. We use the term “guides” for the rest. Guides comprise the final input for the MVEE algorithm—they “guide” it toward better structural approximations. If this step is omitted, all effective atoms are considered guides. It should also be stressed here that the outlier effective atoms (and the actual atoms of residues they represent) are not removed from the analyzed structure. Some of them may (and they often do) end up inside the final ellipsoid and are processed along the guides at the later stages of the EP algorithm.

The strength of this procedure is controlled by the parameter *m* with a default value of 3 (chosen empirically; *m* = 0 means no detection). It denotes the number of medians of the kernel density profile. The first median (*m* = 1) is the median of all its values, the second median (*m* = 2) is the median of all its values below the first median, and so on. The last median is the threshold to isolate the outliers from the guides. This means that some residues in every structure will always be taken as outliers. In globular proteins (e.g., in 1IS9) they are usually distributed on the surface. At *m* = 3, it strengthens the resistance of the ellipsoid bounding procedure against the small external details of the structure and does not let the MVEE algorithm carve too much into it. In non-globular proteins (with a relatively low kernel density, e.g., in 2KKW), it results in the isolation of some residues, mostly located near the chain termini. Put differently, with no significant outliers present, this detection method can be considered stable in terms of ellipsoid size and globularity metrics. However, when such outliers exist (e.g., in 1IIE) and are detected and isolated, the ellipsoid is shrunk, and the metrics of globularity are altered accordingly.

#### 2.3.4. Alignment of the Molecule

The MVEE algorithm returns a quadric form of the bounding ellipsoid, consisting of its center vector *c* and a symmetric, positive-definite matrix *M*. Performing singular value decomposition (SVD) of *M* yields a rotation matrix and inverse squares of lengths of radii of the ellipsoid. The input datasets (i.e., the effective and actual atoms of the protein) can be now brought to the origin by moving *c* to [0,0,0]. They are then rotated in alignment with the coordinate system. It results in the placement of the radii of the ellipsoid on the three principal axes. It is now possible to operate with its simpler, standard equation.

Given point *p* = [*p*_*x*_,*p*_*y*_,*p*_*z*_] and an axis-aligned ellipsoid centered at the origin with radii with lengths *r* = [*r*_*x*_,*r*_*y*_,*r*_*z*_], the value of the standard ellipsoid equation, *ee*(*p*,*r*), is:

(1) $$ee\left(p,r\right)={\left(\frac{p_{x}}{r_{x}}\right)}^{2}+{\left(\frac{p_{y}}{r_{y}}\right)}^{2}+{\left(\frac{p_{z}}{r_{z}}\right)}^{2}$$

*ee*(*p*,*r*) = 0 when *p* = [0,0,0], *ee*(*p*,*r*) < 1 when *p* is inside the ellipsoid, *ee*(*p*,*r*) = 1 when *p* is on the surface of the ellipsoid and *ee*(*p*,*r*) > 1 when *p* is outside the ellipsoid. This concept and the MVEE algorithm generalize to any number of dimensions.

Lengths of the radii of the now-aligned bounding ellipsoid are rounded to nearest integers and increased by 1 Å. For example, if *r_x_* was 34.6 Å, it would be rounded to 35 Å and increased to 36 Å. Note that this 1 Å increase is not performed in the modified version of the EP algorithm (Section 3.1). The radii are needed in the next step (generation of the grid, Section 2.3.5), but at this moment the first two globularity metrics of the EP algorithm can be derived from them: *V* and *T*. *V* is the ellipsoid volume coefficient:

(2) $$V\left(r\right)=\frac{r_{x}\cdot r_{y}\cdot r_{z}}{1000}$$

It is a simple way to join the three radii lengths under one value for size comparison purposes. *T*, on the other hand, is the ellipsoid radii length triangle inequality coefficient:

(3) $$T\left(r\right)=max\left(\frac{r_{x}}{r_{y}+r_{z}};\frac{r_{y}}{r_{x}+r_{z}};\frac{r_{z}}{r_{x}+r_{y}}\right)$$

When *T* < 1, e.g., for *r* = [10,8,6], the protein fits inside a typical triaxial ellipsoid. Its MVEE is a sphere when *T* = 0.5 (*r_x_* = *r_y_* = *r_z_*). However, when *T* ≥ 2, e.g., for *r* = [20,5,5], the molecule is elongated in one dimension, warning about its potentially non-globular structure. The other metrics of the EP algorithm should then be handled with care (i.e., a single helix, such as that in 1AIK:C, may be deemed globular in its sense). There are, however, only a small number of such cases—we found 100 among the 2124 domains [22]. Unsurprisingly, nearly half of them were in the coiled coil SCOP class, *h*. Caution is also advised when processing small structures, i.e., below 75 residues or where min (*r_x_*,*r_y_*,*r_z_*) < 10.

#### 2.3.5. Generation of the Grid

The grid is a rectangular lattice, a set of non-intersecting voxels (1 Å × 1 Å × 1 Å cubes) with centers placed at the integer coordinates. They completely fill the interior of the bounding ellipsoid, which is that part of space where Equation (1) is below 1. For example, if *r* = [40,30,20] (after Section 2.3.4), the grid should contain 100,227 unit Å voxels.

The grid acts as the reference for its subset—for the voxels that represent the actual atoms of the protein. Together, they facilitate an estimation of how much of the ellipsoid is occupied by the molecule. The voxelization happens in the next step (Section 2.3.6).

δ = 1 is the default grid density and voxel size (δ × δ × δ), but one can experiment with other values too. Lower density, e.g., δ = 2, means quicker voxelization and lower RAM consumption at the expense of reduced precision of the measurement of globularity. Conversely, higher density, e.g., δ = 0.5, offers better resolution for smaller structures (peptides, etc.). In any case, one must remember to place the centers of the voxels at the multiples of δ (and to round the radii of the ellipsoid to the multiples of δ before that).

#### 2.3.6. Voxelization of the Protein

The protein is voxelized by finding all voxels from the grid with their centers within the range of the vdW radii of the actual atoms. It is actually the most time consuming part of the computational pipeline of the EP algorithm. Hence, a fast nearest neighbor search is needed. We employ one based on the *k*-d tree structure [47].

NACCESS [48] is the default source of vdW radii definitions in the EP algorithm. They were obtained from the repository of the dr_sasa program [49,50], where they are stored in (residue, atom) pairs. Table 2 from ref. [22] presents our default radii for keys not found in this mapping. In particular, the EP algorithm assumes 1 Å for all instances of hydrogen but ignores them by default. There is a small bias caused by this omission, but it does not significantly impact the measurement of globularity on a large scale.

**Figure 1:**
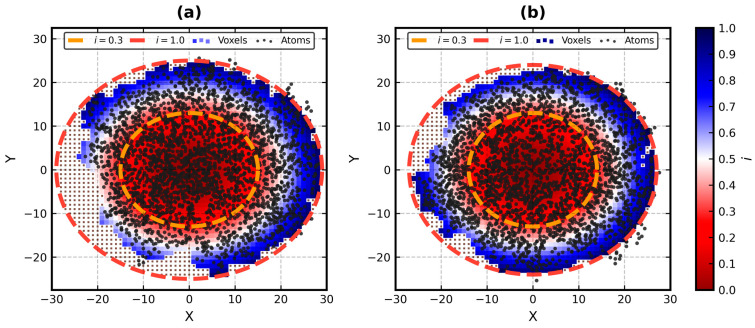
Visualization of the voxelization process using 1IS9 as an example. All 3D coordinates were orthogonally projected onto the XY plane. Outlier detection was disabled on (**a**) and enabled on (**b**), where it resulted in a more befitting ellipsoidal approximation of the shape of this protein. Voxels (here: squares) colored by *i* (the value of Equation (1)) denote that part of the grid that was captured by the nearby atoms of the protein. The unoccupied portion of the grid is shown as the tiny brown dots (note that the three atoms near [−10, −20] on (**a**) are actually above the MVEE). The two dashed ellipses delimit portions of the ellipsoid used to calculate *EI*_0.3_ (amber) and *EI*_1.0_ (red), the two ellipsoid indexes used in the standard measurement of globularity in the EP algorithm.

An example visualization of the generation of the grid, voxelization of the atoms and preparation for the calculation of globularity metrics of 1IS9 is shown in Figure 1.

#### 2.3.7. Ellipsoid Indexes

The grid and its subset, the voxelized protein, are the basis of the main metric of the EP algorithm, the ellipsoid index, *EI*. Let *r* = [*r*_*x*_,*r*_*y*_,*r*_*z*_] be the radii of the bounding ellipsoid (after Section 2.3.4), 0 ≤ *i* ≤ 1 be a user-controlled parameter, *G^i^* = {*g*_1_…*g*_*n*_} be a subset of the grid for which the value of Equation (1) is lower than or equal to *i*, and *H^i^* = {*h*_1_…*h*_m_} be the protein voxels (*m* ≤ *n*; *H^i^* is the subset of *G^i^*) for which the value of Equation (1) is also lower than or equal to *i.* The ellipsoid index at *i*, *EI_i_*, is then calculated as:

(4) $$EI_{i}\left(G^{i},H^{i},r\right)=\frac{m-\sum_{j=1}^{m}ee\left(h_{j},r\right)}{n-\sum_{j=1}^{n}ee\left(g_{j},r\right)}$$

This calculation involves all voxels located in the sense of Equation (1) between the origin (*i* = 0) and *i*, inclusive. Because *H^i^* is the subset of *G^i^*, *EI* can attain values from 0 (no protein voxels present, *H^i^* ≡ *Ø*) to 1 (the protein captures all voxels, *H^i^* ≡ *G^i^*).

Elements of *G^i^* and *H^i^* are weighted in Equation (4) by their corresponding values of 1-Equation (1). This causes voxels located further away from the origin to have lower impacts on the value of the index. There are two reasons for that. First, at the higher values of *i* (e.g., *i* ≥ 0.7), the contribution of voxels near the center of the ellipsoid is not dominated by the contribution of voxels located toward the surface of the ellipsoid. Secondly, it accounts for the fact that even the most globular proteins cannot be perfectly bound in an ellipsoid. There are always some gaps between their atoms and the surface of this shape. Without the weights, these gaps (even the relatively small ones) may cause a sharp drop of the value of *EI* for *i* ≥ 0.7, misrepresenting the status of the external parts of the structure. This phenomenon is less significant for non-globular proteins since their indexes are already low due to the low filling of the volume of their bounding ellipsoids.

#### 2.3.8. Ellipsoid Profile

The ellipsoid profile, *EP*, is the distribution of the values of *EI_i_* for all *i* = [0…1]. Discrete steps of 0.01, yielding 100 data points (*i* on *X* axis, *EI_i_* on *Y* axis), provide a satisfactory default resolution. *EP* displays how the globularity of the protein changes from the center to the surface of its bounding ellipsoid. The higher its values are, the more globular the input protein appears to be. A typical profile decreases as *i* goes past 0.3.

Ellipsoid profiles for the proteins from Table 1 are plotted in Section 3.2.

#### 2.3.9. Globularity Classes

Two empirically chosen ellipsoid indexes, *EI*_0.3_ and *EI*_1.0_, facilitate the standard EP-based classification and comparison of proteins in terms of globularity. The first gauges the status of their interior, while the second gauges the status of the entire molecules:

- the protein appears non-globular (class *N*) when *EI*_0.3_ < 0.3 or *EI*_1.0_ < 0.3;
- the protein appears semi-globular (class *S*) when 0.3 ≤ *EI*_0.3_ < 0.5 and *EI*_1.0_ ≥ 0.3;
- the protein appears globular (class *G*) when *EI*_0.3_ ≥ 0.5 and *EI*_1.0_ ≥ 0.3;
- the protein appears highly globular (class *H*) when *EI*_0.3_ ≥ 0.5 and *EI*_1.0_ ≥ 0.5;
- the protein appears unusual (class *U*, supplemental) when *EI*_0.3_ ≤ *EI*_1.0_;
- the protein appears elongated (class *E*, supplemental) when *T* ≥ 2;

The above labels of globularity classes are formally introduced in this paper. Class *H* is the subclass of class *G*. Members of classes *U* and *E* may belong to any other class.

A semi-globular protein (class *S*) fits well inside the ellipsoid as a whole but does not possess enough atoms to fill its center to attain *EI*_0.3_ ≥ 0.5. It may also signal the opposite—the center of the molecule is filled up with atoms, but their numbers quickly diminish with distance to origin, causing too few of them to reach the surface of the ellipsoid (but still enough for *EI*_1.0_ ≥ 0.3). The semi-globular status can likewise be caused by cavities in the structure which are not severe enough to assign the protein to class *N*.

Unusual proteins exhibit *EI*_0.3_ ≤ *EI*_1.0_. It is a rare situation that becomes progressively more anomalous with the growing difference between the indexes. If a structure is also non-globular, there is a good chance that there is a void in the center of its bounding ellipsoid, that most of its atoms or even entire chains are located toward the surface of this shape. The protein may resemble a sphere, cylinder or torus, just like 3BPD:(A–G) does. So far, we have not encountered a molecule with *EI*_0.3_ ≥ 0.5 and *EI*_1.0_ < 0.3.

Proteins can be compared by scattering them on an *EI*_0.3_ × *EI*_1.0_ map. Since axes of this map are normalized, closeness to the [1,1] corner denotes high globularity, while the distance to this point marks an opposite status. Low globularity is signaled by either index, but they typically change in unison, as corroborated by the *EI*_0.3_ vs. *EI*_1.0_ correlation coefficients for the SCOP superfamily representatives measured close to 0.9 [22].

Ellipsoid index maps for the proteins from Table 1 are plotted in Section 3.2.

#### 2.3.10. Areas under the Profile

The last metric of the EP algorithm is the area under the ellipsoid profile. It is calculated as an *a*/*b* fraction and can attain values from 0 to 1. *a* is the area under the *EP* between two values of *i* (inclusive, measured using the trapezoidal rule) and *b* is the absolute difference between those two values of *i*. This metric estimates how high the profile is running in the *i* × *EI* space within the bounds of the specified *i* range.

Two standard areas are defined. The first is calculated for *i* = [0…0.1] and is written as *|EP|*_0.0–0.1_ or *|EP|*_0.1_ in short. The second is calculated for *i* = [0.1…1.0] and is written as *|EP|*_0.1–1.0_ or *|EP|*_1.0_ in short. Because the shorter forms of those labels are ambiguous, it is recommended to use them only to refer to the standard areas.

The split into two zones of area calculation is motivated by the chaotic behavior of *EP* at *i* ≤ 0.1. Depending on whether the atoms of the protein capture the central voxel at [0,0,0], the profile will start from [0,0] or [0,1] in the *i* × *EI* space. Globularity is promoted by *|EP|*_0.1_ ≥ 0.5 (i.e., the profile should raise if it started from [0,0] and should not fall too much if it started from [0,1]), while *|EP|*_0.1_ < 0.1 hints at the presence of a void in the center of the ellipsoid. On the other hand, *|EP|*_1.0_ is a convenient way to represent the *EI*_0.1_…*EI*_1.0_ series with one value. If *|EP|*_1.0_ < 0.3, the protein is probably non-globular. If *|EP|*_1.0_ ≥ 0.5, the protein is probably globular, maybe even highly globular. However, because it is only an approximation, it is considered a secondary metric to the ellipsoid indexes.

### 2.4. Tools and Websites

Three-dimensional images of the proteins were rendered with PyMOL [51,52] and PyVista [53]. PyVista is a streamlined Python interface to the Visualization Toolkit (VTK) [54]. Charts were plotted using the Matplotlib library [55]. Our software modules employ the state-of-the-art open-source Python libraries for scientific computation [56,57]. Online access to the ellipsoid profile algorithm, the fuzzy oil drop algorithm and related bioinformatics tools is possible at the http://fod.cm-uj.krakow.pl web server.

## 3. Results and Discussion

The Results section is split into two parts. In the first part (Section 3.1, Section 3.2 and Section 3.3) we describe an enhanced structural outlier detection subroutine for the ellipsoid profile algorithm and discuss the outcome of its application to the 2124 SCOP domain representatives at the superfamily level (i.e., the modified ASTRAL 2.08 subset—see Section 2.1). In the second part (Section 3.4) we first share instructions for how to extract a non-redundant set of representatives of the biological assemblies from the PDB, after which we study how the improved EP algorithm handles the 3594 of them.

### 3.1. The Improved Ellipsoid Profile Algorithm

We have recently [26] introduced to the calculation pipeline of the fuzzy oil drop (FOD) model an alternative approach to the alignment of the input protein with the axes of the coordinate system. Such alignment procedure is needed to properly assign values of theoretical hydrophobicity to the residues via 3D Gaussian function. Our modification is based on principal component analysis (PCA) and the confidence ellipsoid method.

#### 3.1.1. The Principal Component Analysis

PCA [58,59] is a simple yet powerful technique that solves the eigenvector/eigenvalue problem to find an optimal linear transformation of its input dataset (*n* samples—observations, each described by *d* numeric variables—properties) to a new system where the mean is zero and the variance of all variables, now uncorrelated, is maximized [60]. If we treat those variables as Cartesian coordinates, PCA turns into an optimizer that fits a *d*-dimensional ellipsoid to a cloud of points by minimizing their orthogonal distance to its axes [27]. This, in particular, allows alignment of those points with the principal axes of the coordinate system in decreasing order of variance (highest on X axis, then on Y axis, etc.). PCA is efficiently calculated via SVD. It works in any number of dimensions.

#### 3.1.2. The Confidence Ellipsoid

The variables (e.g., the coordinates) of the input samples (e.g., the effective atoms) aligned via PCA are independent—they have zero covariance. Assuming that they are normally distributed, Equation (1) can be turned into a sum of squares of normalized variables. To attain that, [*r*_*x*_,*r*_*y*_,*r*_*z*_] need to be set to [*σ*_*x*_,*σ*_*y*_,*σ*_*z*_], the standard deviations in each dimension. The values of Equation (1) should then follow the χ^2^ distribution with *d* = 3 degrees of freedom [28]. This concept generalizes to any number of dimensions. Here it opens the possibility to estimate the size of an ellipsoid fit via PCA to a protein [26].

The size of an axis-aligned confidence ellipsoid that splits the observations (points) into internal (model) and external (outliers) subsets can be inferred from [*σ*_*x*_,*σ*_*y*_,*σ*_*z*_] and a scaling coefficient *s*. The value of *s* depends on the confidence level *P* (0 < *P* < 1) selected by the user. Increasing the value of *P* increases the probability for each point to fall inside the confidence ellipsoid [28]. Point [*x,y,z*] lies on its surface when:

(5) $${\left(\frac{x}{\sigma_{x}}\right)}^{2}+{\left(\frac{y}{\sigma_{y}}\right)}^{2}+{\left(\frac{z}{\sigma_{z}}\right)}^{2}=s=\chi_{\left(P,3\right)}^{2}$$

To solve for the lengths of the radii of this ellipsoid, one should use [*x*,0,0], [0,*y*,0] and [0,0,*z*] in the above equation, resulting in *x* = *σ*_*x*_*√s, y* = *σ*_*y*_*√s* and *z* = *σ*_*z*_*√s*. It is needed because PCA returns only the direction vectors of the radii (i.e., the principal components). It also means that the outcome depends on *P*. The choice of its value may be non-trivial.

#### 3.1.3. The FOD–PCA Algorithm

The altered workflow of the FOD algorithm was named FOD–PCA [26]. In ref. [26] we deemed it optimal for the purpose of alignment of the molecule, as in comparison to the baseline FOD model, it is faster, achieves a diagonal covariance matrix of the effective atoms and automatically aligns them with respect to structural symmetry, absolving the user from supervising this procedure (the baseline approach may require manual input of the main axis of symmetry). EP and FOD share the definition of an effective atom. FOD uses effective atoms to represent residues in space in a coarse-grained manner.

Once the effective atoms of the protein have been aligned (i.e., translated to the origin and rotated), the size of the drop must be calculated. The drop of the FOD model is an axis-aligned ellipsoid that encompasses the input structure. The lengths of its radii, each divided by three (i.e., the three sigma rule), yield the standard deviation parameters for the 3D Gaussian function, which, in turn, is used to calculate the theoretical hydrophobicity (i.e., the *T* distribution). In the baseline FOD algorithm these radii are obtained from the coordinates of the effective atoms located furthest away from origin in each dimension. In the FOD–PCA modification they are obtained from the same coordinates but also from the lengths of the radii of a confidence ellipsoid fit to the effective atoms at *P* = 0.75 (those radii are finally extended in both FOD versions by 9 Å, but that is irrelevant here). Owing to FOD-PCA, the bounding of the protein in the drop becomes less susceptible to structural outliers and more closely connected to the distribution of all effective atoms, the property that is eventually encoded in the *T* distribution. It is also possible to employ the PCA-based alignment together with the baseline drop size selection method. The service at http://fod.cm-uj.krakow.pl website allows that.

One may notice that the above workflow is similar to what the EP algorithm tries to achieve. In fact, it shares a common goal with the FOD model to some extent. The difference is that the EP algorithm can be more decisive in terms of the ellipsoidal representation of the molecule, whereas a subtle balance needs to be maintained with the size of the drop of the FOD model due to the strong dependence of theoretical hydrophobicity on it. The FOD-PCA modification is not aiming to throw all outlying, exposed to the solvent fragments of the chain out of the drop, but to reduce their influence on its size. It is particularly useful when those fragments are small or otherwise should not be removed a priori by hand. An additional subroutine, enabled at user’s discretion, would be needed to remove any problematic outliers or report their existence for manual handling.

#### 3.1.4. The Problem with the Kernel Density

We realized that in the context of the measurement of the globularity of proteins, the PCA and confidence ellipsoid tandem can be a powerful ally for the outlier handling subroutine of the EP algorithm. Recalling Section 2.3, there are three goals to achieve here:

1 decrease susceptibility of the MVEE to the features of protein’s molecular surface;
2 isolate significant outliers from the guides in structures where outliers are present;
3 do not significantly impact the EP algorithm’s metrics if outliers are not present.

We also discovered that the current approach based on medians of kernel density runs into trouble on some occasions at the second point above. For example, a cluster of effective atoms at the end of an exposed chain fragment can prevent some of them from being classified as outliers, leaving them “disconnected” from the rest of the molecule. Offsetting it by decreasing the value of *m* can cause too much of the protein to be isolated. Perhaps an alternative method for the selection of the low density regions in the structure could fix this, but since PCA and confidence ellipsoid work well for the FOD algorithm, we decided to give them a try in the context of the measurement of globularity too, checking how capable they are as a potential replacement for the KDE-based workflow.

#### 3.1.5. The PCA-Based Outlier Detection Subroutine

While experimenting with various values of *P* in Equation (5) we came to the conclusion that there is no universal confidence level that is robust against the many shapes of protein structures. A *P* that is low enough to capture all outliers (such as the aforementioned clusters at the end of a chain) may also be low enough to carve too much into the molecule. Conversely, a *P* that is high enough to not carve too much into the molecule may also fail to capture even the most evident outliers. Naturally, the same applies to the KDE-based approach and its *m* parameter. However, because PCA is fast (i.e., much faster than KDE, permitting its application even to the actual atoms of the protein), we tried an iterative approach which involves running it a few times and trimming the guide effective atom set with each step toward the dense “main” portion of the structure.

**Figure 2:**
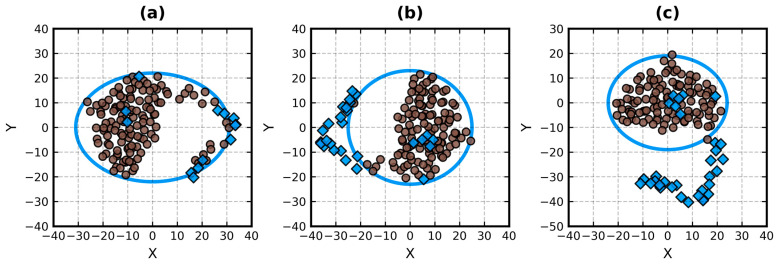
Three rounds of structural outlier detection in 4B0H:B based on the PCA plus confidence ellipsoid tandem using *r* = 3 (number of rounds) and *P* = 0.9 (confidence level). Markers denote the 143 effective atoms orthogonally projected onto the XY plane (diamonds—outliers, circles—guides). The guides would be used in the bounding of this chain in the MVEE (i.e., the blue ellipse) if the detection subroutine terminated at a given round. In each round, the algorithm isolates more outliers from the guides: (**a**) 13 outliers vs. 130 guides (*r* = 1), (**b**) 27 vs. 116 (*r* = 2), (**c**) 32 vs. 111 (*r* = 3).

At the beginning, all effective atoms are considered guides and there are no outliers. In each round (i.e., in each loop iteration), the current set of guides is aligned with the axes of the coordinate system via PCA and its outliers are isolated via Equation (5). This process repeats until either all rounds have passed or all guides have stayed inside the confidence ellipsoid (i.e., no change). Its visualization for 4B0H:B is shown in Figure 2.

We chose *r* for the label of the round number parameter and found 3 to be its good default value. Users are encouraged to experiment with it. For instance, at *r* = 4 that lone guide effective atom below the bottom arc of the MVEE on Figure 2c can be marked as an outlier. However, it results in the decrease in *r*_*y*_ by 2 Å and a small change in the globularity metrics. Setting *r* = 0, just like setting *m* = 0 in the KDE-based method, bypasses the outlier detection subroutine, sending all effective atoms as guides to the MVEE algorithm. *P* ≥ 1 can be treated like *r* = 0 since it denotes an infinite confidence ellipsoid.

The recommended value of *P* is 0.9 (*s* ≈ 6.251, √*s* ≈ 4.108). On the basis of the results of the bounding of structures from Table 1 (n.b., their visualization in the style of Figure 2 for *r* = 3 and *r* = 0 is shown in Supplemental File S2—Figures S7–S12), 0.9 appears to be a dependable default level of confidence. It isolates the outliers in a predictable manner and may finish the procedure before *r* is reached. For example, it permits at most four iterations with 4B0H:B and at most three with 3BPD:(A–G). Using *r* as a safeguard is still needed, as in some cases the guide set may keep shrinking. It happens in 1DIV, which is reduced by *P* = 0.9 and *r* = ∞ (i.e., no limit) to the complex of the C-terminal domains. This coincidentally provides another hint about the non-globular structure of this protein.

Because of the median-based threshold of the kernel density, the KDE-based EP algorithm always marks some of the effective atoms as outliers (≈12.5% at *m* = 3; whether they fall in or out of the MVEE is a different story), while the shape of the protein influences their location: from convergence in the outlier regions (e.g., as in 1IIE) to scattering on the surface (e.g., as in 1IS9). Conversely, the PCA-based approach seems to use more guides in globular proteins, e.g., 354/358 vs. 313/358 in 1IS9. To balance that, we opted to not increase the rounded MVEE radii by δ with it (compare this with Section 2.3.4).

If one finds *r* = 3 too strong, using *r* = 1 or *r* = 2 is a viable alternative to it.

### 3.2. Improved Ellipsoid Profile of the Example Proteins

The EP algorithm enhanced with the PCA-based outlier detection was administered to the selected fragments of the six proteins from Table 1. Their metrics of globularity are presented in Table 2 (*r* = 3) and Table 3 (*r* = 0). The visualization of their bounding and voxelization is shown in Supplemental File S3—Figures S13–S18, while the corresponding measurements taken with the KDE-based method are given in Tables 3 and 4 of ref. [22].

3BPD:(A–G) is one of the two identical biological assemblies from 3BPD. Together with 1IS9 it represents proteins that can be easily put in an ellipsoid and do not possess significant outliers. 4B0H:B and 1IIE exhibit such outliers. The first is a chain, the second is a trimer. Chains B and C are longer than chain A in 4B0H, but chain A is included in the official ASTRAL superfamily representative subset, which made us analyze it previously [22]. The KDE-based outlier detection has trouble with the isolation of the entire “arm” in chains B and C. Lastly, 2KKW and 1DIV are two non-globular proteins with no disordered chain fragments but with a spread/loose conformation that puts them in class *N*.

From now on in this and the next subsection, “PCA” is used as the shorthand for the improved EP algorithm utilizing the new, PCA-based outlier detection, whereas “KDE” denotes the original EP algorithm that employs the KDE-based outlier detection.

The lengths of the radii of the bounding ellipsoid were similar in proteins without outliers: 27 × 24 × 23 (*V* = 14.9) with PCA vs. 28 × 24 × 22 (*V* = 14.8) with KDE for 1IS9 and 39 × 39 × 23 (*V* = 35) with PCA vs. 41 × 40 × 21 (*V* = 34.4) with KDE for 3BPD:(A–G). Their ellipsoid indexes were likewise constant in terms of classification, 1IS9 stayed in class *H* and 3BPD:(A–G) stayed in classes *N* and *U*, but their values dropped with PCA by 0.02≈0.04. The value of |*EP*|_1.0_ for 3BPD:(A–G) also went below 30%, which is a more befitting reflection of its non-globular status. Membership of class *U*, |*EP*|_0.1_ ≈ 0 and a high *EI*_1.0_–*EI*_0.3_ difference strongly hint about the toroidal shape of this molecule.

The situation changed with the presence of actual outliers: 24 × 19 × 13 (*V* = 5.9) with PCA vs. 33 × 24 × 19 (*V* = 15) with KDE for 4B0H:B and 22 × 22 × 18 (*V* = 8.7) with PCA vs. 23 × 23 × 22 (*V* = 11.6) with KDE for 1IIE. A stronger reduction of the size of the ellipsoid by PCA is noticeable in 4B0H:B, for which KDE at *m* = 3 yielded the following metrics of globularity: *EI*_0.3_ = 0.284, *EI*_1.0_ = 0.247, *|EP|*_0.1_ = 0.485 and *|EP|*_1.0_ = 0.277. This high difference was caused by the aforementioned outlier detection issues in this chain. The PCA-based version of the EP algorithm is not encumbered by them. On the other hand, both methods produced comparable globularity metrics and identical globularity classification (class *H*) for 1IIE. They correctly removed all three disordered chain fragments.

Reduction of the lengths of the ellipsoid radii was also observed in the non-globular molecules: 62 × 23 × 19 (*V* = 27.1) with PCA vs. 61 × 36 × 22 (*V* = 48.3) with KDE for 2KKW and 45 × 35 × 22 (*V* = 34.6) with PCA vs. 51 × 45 × 20 (*V* = 45.9) with KDE for 1DIV. When a protein such as the Alpha Synuclein has virtually no tertiary structure, its residues exhibit a relatively low kernel density (lowest in the unstructured regions). Thus, the KDE-based approach marked just a few residues near the C-terminus of 2KKW as outliers. On the other hand, PCA isolated about half of that region. The ellipsoid indexes correctly stayed below 0.1 (0.02≈0.04 PCA vs. KDE difference). It can be explained by the fact that in the NMR conformer 1 of 2KKW, the C-terminal region is perpendicular to the plane formed by the two helices that constitute the main body of this protein (M1–L100). This stimulates PCA to work toward marking it wholly as an outlier. In fact, using *r* = ∞ reduces the guide effective atom set to the D2–Q99 region (48 × 23 × 8, *V* = 8.8), but it remains non-globular (|*EP*|_1.0_ = 0.148) due to the space between the helices. Interestingly, the M1–L100 region is what one would send to the FOD algorithm for hydrophobic core analysis. The treatment of about half of the M1–Q55 domains of 1DIV as outliers by PCA resulted in a noticeable increase in *EI*_0.3_ versus KDE by ≈0.09 and a small reduction of *EI*_1.0_ by ≈0.03. The protein remained seen as non-globular. As mentioned previously, *P* = 0.9 and *r* = ∞ leave only its R56–K149 domain complex as the guide set. This complex is also highly globular (*EI*_0.3_ = 0.511, *EI*_1.0_ = 0.529). However, also interestingly, at *r* = 3 the entire molecule is bound in the MVEE in a way that follows its cyclic symmetry more closely than during bounding at *r* = 0 (Figure S12 in Supplemental File S2).

**Figure 3:**
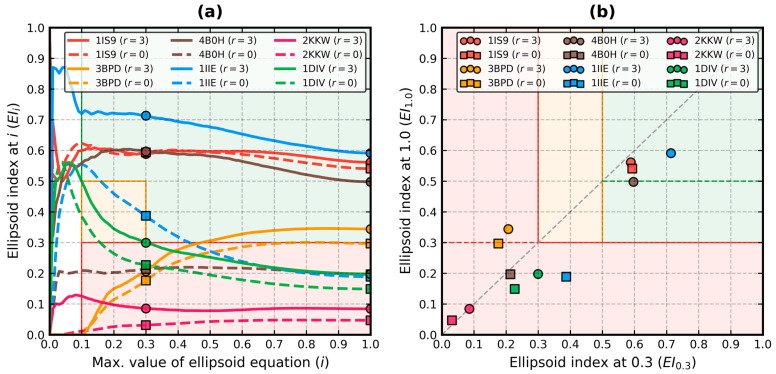
Ellipsoid profiles (**a**) and ellipsoid indexes (**a**,**b**) of proteins from Table 1 calculated with the PCA-based outlier detection on (*r* = 3, solid lines, circle markers) and off (*r* = 0, dashed lines, square markers). Coordinates of markers on (**b**) corresponds to Y axis values of matching markers on (**a**). The proteins are labeled here by their PDB codes, but the fragments actually passed to the EP algorithm are the same as in Table 2. The red dashed line on (**b**) identifies structures exhibiting *EI*_0.3_ < 0.3 and *EI*_1.0_ ≥ 0.3. The green dashed line is the boundary of class *H.* Structures above the grey dashed diagonal are in class *U*. The colored backgrounds act as visual cues for the classification of globularity: red—class *N*, orange—class *S*, green—class *G*. The white zone on (**a**) is for *|EI|*_0.1_.

The ellipsoid profiles and indexes of the example proteins calculated with the PCA-based EP algorithm are presented in Figure 3. The corresponding data from its KDE-based counterpart are plotted in Figures 5 and 6 of ref. [22]. In both approaches 3BPD:(A–G) is located in the rare zone within classes *N* and *U* where *EI*_0.3_ < 0.3 and *EI*_1.0_ ≥ 0.3.

The ellipsoid index difference between PCA and KDE without outlier detection (i.e., at *r* = 0 and *m* = 0) was measured in the second significant digits. It was largest in 1IS9 and 3BPD:(A–G), where *EI*_0.3_ dropped by ≈0.02 and *EI*_1.0_ raised by ≈0.01. It is the effect of the shortened ellipsoid radii (i.e., no extension by δ). 1IS9 also became the only structure in this set with all its metrics suggesting (high) globularity at both *r* = 3 and *r* = 0. Previously, 3BPD:(A–G) had *EI*_1.0_ slightly above 0.3—now it was a bit below it. The requirement that a structure that does not possess significant outlier fragments should exhibit similar globularity metrics regardless of the use of outlier detection is maintained by PCA.

The *|EI|*_1.0_ difference (Table 2 and Table 3) was 0.009 for 1IS9, 0.038 for 3BPD:(A–G), 0.35 for 4B0H:B, 0.367 for 1IIE, 0.048 for 2KKW and 0.061 for 1DIV. Area loss higher than 30% seems to signal the presence of the outliers. However, since they are strongly protruding here, 15% could be enough to capture their less outstanding variants. The globularity classification should also change due to the enlargement of bounding ellipsoid. It happened to 4B0H:B and 1IIE—they were demoted from class *G* at *r* = 3 to class *N* at *r* = 0.

Lastly, the analysis of the dynamics of the profile may unveil additional information about the protein. For instance, 1DIV experienced a sharp drop of its *EP* near *i* = 0.1 at both *r* = 3 and *r* = 0. It was caused by the helix that connects the two domains in each chain and the spread conformation of the complex of those chains.

### 3.3. Improved Ellipsoid Profile of the Domain Superfamilies

To validate the performance of the improved EP algorithm on a large scale, we applied it to the 2124 protein domain superfamily representatives from the modified SCOP 2.08 ASTRAL compendium (Section 2.1). We replicated our previous experiment [22]. The improved EP algorithm was run twice on each domain, with outlier detection (*r* = 3) and without it (*r* = 0). The results were again split between the eight SCOP classes (*a–h*). They are visualized via *EI*_0.3_ × *EI*_1.0_ maps (i.e., in the form of Figure 3b) in Supplemental File S4—Figures S19–S26, while the summary of the globularity metrics is given in Table 4 (*r* = 3) and Table 5 (*r* = 0). The values of those metrics for all individual domains are stored in Supplemental Files S5 (*r* = 3) and S6 (*r* = 0) in tab-separated textual table format.

**Figure 4:**
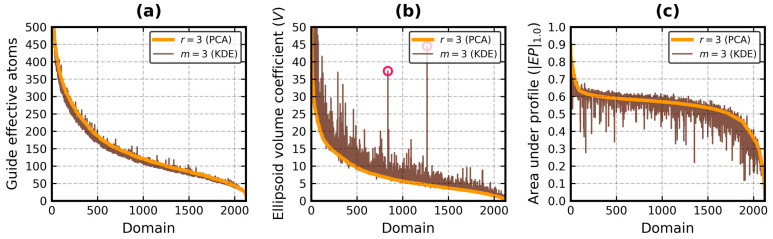
Comparison of selected globularity metrics returned by the modified (PCA) and original (KDE) versions of the EP algorithm for the 2124 SCOP domain superfamily representatives with outlier detection turned on: (**a**) number of guides, (**b**) *V* coefficient and (**c**) *|EP|*_1.0_. The results are sorted in the decreasing order of value of the metrics for the PCA-based algorithm.

**Figure 5:**
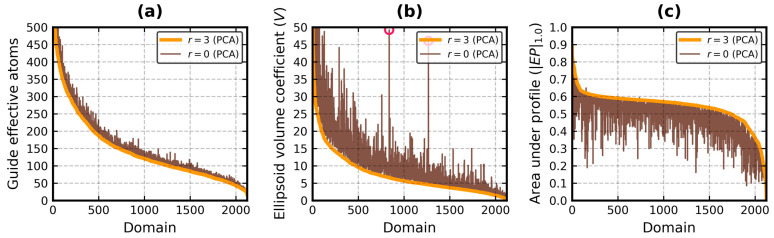
Comparison of selected globularity metrics returned by the modified (PCA) version of the EP algorithm for the 2124 SCOP domain superfamily representatives with outlier detection turned on (*r* = 3) and off (*r* = 0): (**a**) number of guides, (**b**) *V* coefficient and (**c**) *|EP|*_1.0_. The results are sorted in decreasing order of the value of the metrics for *r* = 3.

Figure 4 and Figure 5 facilitate a visual comparison of the values of three metrics of the EP algorithm: number of guide effective atoms, *V* coefficient and *|EP|*_1.0_. Figure 4 is for *r* = 3 and *m* = 3 (i.e., PCA vs. KDE), while Figure 5 is for *r* = 3 and *r* = 0 (i.e., PCA vs. PCA).

It is confirmed that the EP algorithm enhanced with PCA produced a higher number of guides, ≈93% on average (*σ* ≈ 5%) in this database, while its KDE-based version delivered a stable ≈88% at *m* = 3 (Figure 4a). However, it is not a universal rule, as PCA employed less guides than KDE 308 times. It can be primarily attributed to the stronger outlier detection. Two SCOP classes with the lowest guide averages were *f* and *h*, the membrane and coiled coil proteins. The difference between PCA and KDE in terms of number of guides in the entire database ranged from −51 to 62, ≈8 on average (*σ* ≈ 10.5).

The bounding ellipsoids produced with PCA were generally smaller than those obtained with KDE (Figure 4b). Their average *V* coefficient for all domains was measured at 7.4 (*σ* = 7.2), whereas the same average for KDE was equal to 9.6 (*σ* = 10.4). It should be noted, however, that *V* can rapidly change even with small changes to the ellipsoid radii lengths. For instance, it is 32.6 for 34 × 32 × 30 and 35.8 for 35 × 34 × 31.

The two tall peaks in the middle of Figure 4b and Figure 5b (marked by pink circles) are domains h.4.11.1 (Chemotaxis phosphatase CheZ), represented by chain Z from the protein with PDB code 1KMI [61], and a.137.10.1 (Stathmin), represented by chain E from the protein with PDB code 3RYC [62]. Both are made of long helices and feature a ≈90° bent at the end of the chain. 1KMI:Z has over 30 residues not found during the crystallographic experiment, while 3RYC:E has two beta strands at the N-terminus. KDE had trouble with handling those parts. PCA swiftly isolated them from the rest of the chain.

The PCA-based and KDE-based versions of the EP algorithm returned similar globularity metrics with outlier detection turned on (Figure 4c). The average value of *|EP|*_1.0_ was 0.54 (*σ* = 0.09) with PCA and 0.53 (*σ* = 0.11) with KDE. It was less than 0.5 in class *e* with PCA and in classes *e* and *f* with KDE. The conclusion that domains of SCOP generally fit well inside the effective atom MVEEs remains. The average values of *EI*_0.3_ and *EI*_1.0_ were higher or equal to 0.5 and 0.4, respectively, with both approaches for every SCOP class. PCA even achieved an overall *EI*_1.0_ average of 0.5 (it was 0.47 with KDE). The average index difference between the two methods for all domains was 0.0 (*σ* = 0.05) for *EI*_0.3_ and 0.03 (*σ* = 0.05) for *EI*_1.0_. It ranged from -0.01 to 0.07 for the individual SCOP classes.

While the overall value shift of the ellipsoid indexes was not significant, it was apparently enough for PCA to put 1824 domains in class *G*, with 1306 of them also in class *H*, whereas KDE did that to 1735 (class *G*) and 965 (class *H*) domains, respectively. For classes *S* and *N* these numbers were equal to 213 vs. 211 and 87 vs. 178 (PCA vs. KDE). The two approaches agreed 942 (*H*), 1709 (*G*), 117 (*S*) and 85 (*N*) times. These results, in particular for classes *N* and *H*, suggest that the PCA-based method leans toward higher globularity scores. However, only 17 of the 364 domains switched to class *H* from classes other than *G*. It happened due to improved outlier handling, for example in the c.106.1.0 domain (SurE-like), which was represented by chain A from the protein with PDB code 2WQK [63]. The other domains had *EI*_1.0_ right below 0.5, which allowed them to leap over this boundary. An Oncogene products domain (SCOP code b.63.1.1) from the structure with PDB code 1A1X [64] is a good example of this phenomenon. Its *EI*_1.0_ increased from 0.468 with KDE to 0.523 with PCA. Improvements to the outlier detection process were also corroborated by the halving of the number of members of class *N*.

There was no significant discrepancy between PCA and KDE with outlier detection turned off. The *V* coefficients became larger but remained in similar distance from each other (μ = 11.2 and *σ* = 12.3 with PCA vs. μ = 13.1 and *σ* = 13.6 with KDE). The average differences between pairs of globularity metrics (e.g., *EI*_0.3_ with PCA vs. *EI*_0.3_ with KDE) ranged from -0.02 to 0.03 for every SCOP class (*σ* ≤ 0.4). It was caused by the abandonment of the extension of the ellipsoid radii by δ. The classification of the domains was also found to follow the schema described in the last paragraph: 466 (PCA) vs. 312 (KDE) in class *H* (311 agreements), 1483 vs. 1520 in class *G* (1477), 326 vs. 247 in class *S* (244) and 315 vs. 357 in class *N* (314). Only this time, the class change happened only to domains that were close to the class boundary. For instance, the 43 of them that fell from class *G* to class *S* had average *EI*_0.3_ at *m* = 0 equal to 0.508 (*σ* = 0.006), and the 39 that ascended from class *N* to class *S* had average *EI*_1.0_ at *m* = 0 equal to 0.294 (*σ* = 0.005). Such proteins can be thought as having “fluid” globularity classification—prone to be easily changed.

When outlier detection was enabled, the numbers of atypical results increased with PCA from 79 to 108 in class *U* (63 common) and from 100 to 127 in class *E* (98 common). Disabling it changed these numbers to 69, 104 and 69 for class *U* and to 80, 92 and 80 for class *E*. However, to join class *U*, a structure only needs to exhibit *EI*_0.3_ ≤ *EI*_1.0_, so it may fluctuate in and out of this class if it is located near the *EI*_0.3_ = *EI*_1.0_ diagonal on the map.

There is only one last pair of results left to compare: *r* = 3 vs. *r* = 0 (*m* = 3 vs. *m* = 0 was conducted in ref. [22]). The number of guides (Figure 5a) was already analyzed. Skipping the outlier detection step increased *V* by 3.8 on average (*σ* = 6.3, Figure 5b), the most in class *e* (μ = 11.1, *σ* = 13.9). It also decreased *EI*_0.3_ (μ = −0.05, *σ* = 0.07), the most in classes *f* and *h* (μ ≈ −0.11, *σ* ≈ 0.11). The average and standard deviation of the overall *EI*_1.0_ difference were equal to -0.08 and 0.06, respectively. The highest shifts were again observed in classes *f* and *h* (μ ≈ −0.11, *σ* ≈ 0.09). *r* = 0 caused |*EP*|_1.0_ to drop on average by 0.06 (*σ* ≈ 0.06, Figure 5c). In total, 466 domains belonging to class *H* at *r* = 3 remained there at *r* = 0, 1475 remained in class *G*, 85 remained in class *S*, 86 remained in class *N*, 69 remained in class *U* and 89 remained in class *E*.

Lastly, we estimated the number of domains that may possess significantly outlying fragments. Following the criteria from Section 3.2, switching from *r* = 3 to *r* = 0 should change the globularity class to worse (*G* to *S*, *G* to *N* or *S* to *N*) and the |*EP*|_1.0_ difference should reach 15% or more. It happened to 186 domains, the most in classes *d* (48) and *a* (41), the least in *e* (8) and *g* (11). However, when expressed as a fraction of the number of domains in those classes, *f* and *h* became the leaders, both at ≈21% (27/130 and 14/66). 65 domains shifted from class *G* to class *S*, 37 shifted from *S* to *N* and 84 shifted from *G* to *N*. The last number is the most probable count of domains with significant outliers. 2WQK:A fits perfectly in this scheme with its domain-swapped chain fragments.

### 3.4. Improved Ellipsoid Profile of the Biological Assemblies

After the successful application of the improved EP algorithm to the SCOP domain superfamily representatives, we decided to step up the challenge and use it in the survey of the landscape of globularity of the proteins, but at the quaternary structure level. Since SCOP does not maintain this kind of information, a new dataset had to be constructed.

#### 3.4.1. Creation of the Database

An imposing majority of protein structures deposited in the PDB was obtained via X-ray diffraction [65]. The experiment produces crystallographic interfaces that must be distinguished from the biologically relevant interfaces. Put differently, some asymmetric units must be joined into biological assemblies, and some biological assemblies must be extracted from asymmetric units. Some biological assemblies are also equal to the asymmetric units. In any case, it is a non-trivial task and many computer methods were developed to handle it [37,38]. One such tool, PISA (protein interfaces, surfaces and assemblies) [66] is employed by the PDB for automated quaternary structure matching.

A biological assembly is the largest functional form of a protein (i.e., its quaternary structure) that was captured during the experiment [37,38]. As of today, all PDB structures should have at least one such assembly (“biomolecule”) defined and each of their chains should be assigned to at least one of the biomolecules. When depositors perform this assignment themselves, it appears in the REMARK 350 records of the PDB file under “author determined biological unit” along with any additional hits from PISA (“software determined quaternary structure”). Symmetry operators needed to rebuild the assembly from the asymmetric units are provided in the same location. If there is no need to do that, the transformation is simply an identity matrix with a zero translation vector.

The quaternary structures denoted by the biological assemblies from the PDB files constitute a reasonable target group for the EP algorithm, but the validation of the correctness of their annotation was beyond the scope of this paper. It was also necessary to avoid duplicates and to be able to somehow classify and cluster those assemblies. Hence, we decided to consult an established resource, the 3DComplex database [39,40]. It performs all the above tasks with manual curation—it validates, classifies and clusters the biological assemblies from the PDB in terms of their structure and sequence. Contrary to its name, it also processes monomeric proteins. All data are available to the public.

3DComplex stores its biological assemblies in a forest. The complexes are encoded and clustered at the top of the hierarchy via graphs of non-bonded chain contacts (“QS topology”). They are also assigned to symmetry classes (*C*_2_, *D*_3_, etc.). The clusters are split on the next level on the basis of domain superfamily architecture (“QS family”). The trees divide further down with increasing levels of uniqueness of the sequence.

We chose to work with the clusters at the QS family level. They are diverse enough and possess a low probability for inter-cluster structural similarities. The corresponding file NRX_0_5_topo_label_clusters.txt from the 3DComplex website (version 6, bottom-up hierarchy) defined 11,582 such clusters containing 167,079 assemblies. Each cluster had a representative chosen by 3DComplex on the basis of its crystallographic resolution.

We decided to add another criterion to the choice of cluster representatives. In addition to good resolution, the assemblies should also possess a low number of model errors, understood here as the missing (i.e., unresolved) or unreliable (i.e., zero occupancy) residues. This information is stored in the PDB files in REMARK 465 and 475 records. We devised the following formula to combine these factors into one scoring function:

(6) $$R^{2}+{\left(E=\sum_{i=1}^{c}\frac{m_{i}+u_{i}}{\sqrt{l_{i}}}\right)}^{2}$$

The representative of a cluster had to have the lowest score in that cluster—it had to be closest to [0,0] in the *R* × *E* space. Symbols in this equation have the following meaning:

- *R* and *E* are the crystallographic resolution and error coefficient, respectively;
- *m_i_* is the number of residues in REMARK 465 for the *i*-th chain in the assembly;
- *u_i_* is the number of residues in REMARK 475 for the *i*-th chain in the assembly;
- *l_i_* is the length of the sequence (SEQRES records) of the *i*-th chain in the assembly;
- *c* is the total number of chains in the reconstructed assembly (i.e., after the application of all required symmetry operators from REMARK 350, between 1 and 60).

√*l* permitted penalization of errors in both small and large assemblies, keeping those errors relevant along *R*, while squares of the summation terms discouraged *E* from dominating the resolution. Together, they allowed us to avoid the low quality models.

However, before the above selection was made, assemblies determined only by the software (e.g., by PISA) or involving nucleic acids were discarded. To promote the high accuracy of complex symmetry information, assemblies still unprocessed by 3DComplex (i.e., with “?” in the corrected_sym column), known or suspected to be erroneous (i.e., with “YES”/“PROBYES” in the pdb_error column) were also removed. For the same reason, we chose to omit the 5207 possibly problematic structures for which 3Dcomplex corrected the PDB’s symmetry class or the number of subunits.

Additionally, before the application of Equation (6), custom SCOP domain fingerprints were compiled for all assemblies (see next paragraph). Those with chains without at least one SCOP entry or which contained domains from SCOP classes *i*, *j* or *k* were removed. Class *l* (terminal artifacts) was permitted but not included in the fingerprints. It was performed as a precaution against structural duplicates and had the largest impact on the size of the database. For this reason, it also affected the selection of assembly cluster representatives via Equation (6). When representatives of multiple clusters had an identical domain fingerprint at the SCOP superfamily level, their clusters were merged into one cluster, which was then represented by its member that had the lowest value of Equation (6). There were 330 such clashes involving a total of 793 assemblies.

The assembly fingerprints were written in the following way: (d.99.1.1,d.100.1.1)*2. This one corresponds to 1DIV, where each chain (*2) is composed of two domains from d.99.1.1 and d.100.1.1 families. (d.99.1,d.100.1)*2 was its fingerprint at the superfamily level. Domain family codes were stored in order of sequence and multi-segment domains (e.g., spanning residues 1−100 and 200−300) were only captured the first time they were encountered. (a.26.1.2)*2+(b.1.1.1,b.1.1.2)*1 is an example of a fingerprint of a heterotrimer where two chains that are identical in the sense of SCOP domain families form a complex with a third chain that is composed of two other domains. Because this encoding had to be invariant to chain ID, the order of terms between the + signs did not matter, hence (a.26.1.2)*2+(b.1.1.1,b.1.1.2)*1 equaled (b.1.1.1,b.1.1.2)*1+(a.26.1.2)*2.

3594 biological assemblies from 3577 PDB structures comprised the final database. They represented a total of 136,608 biological assemblies from the PDB. A total of 638 clusters had only one member. The average values of *R*, *E* and of the square root of Equation (6) were 2 (*σ* = 0.56), 1.77 (*σ* = 3.93) and 3.08 (*σ* = 3.66), respectively. *E* was zero (i.e., no missing or unreliable residues) in 842 assemblies.

#### 3.4.2. Analysis of the Database

The biological assemblies from the database were divided into subsets depending on their symmetry class, number of chains, domain stoichiometry (i.e., whether all chains had the same domain fingerprint at the family level), and the number of domains in the chains (i.e., whether there was one domain in all chains or more than one domain in some of the chains). The number of possible subsets was high, but because some of them would have less than 10 members, only relevant splits were made. This resulted in the creation of following 15 assembly groups (characters in parentheses denote their labels):

- asymmetric—monomers with one (A1o) or many domains (A1m), dimers (A2), trimers (A3) and complexes of four or more chains (A4+);
- cyclic—homomers with *C*_2_ symmetry and one (C2o=) or many domains (C2m=), heteromers with *C*_2_ symmetry and one (C2o≠) or many domains (C2m≠), complexes with *C*_3_ symmetry (C3) and complexes with *C*_4_ or higher order symmetries (C4+);
- dihedral—complexes with *D*_2_ (D2), *D*_3_ (D3) and *D*_4_ or higher order symmetries (D4+);
- complexes with either tetrahedral, octahedral or icosahedral symmetries (TOI).

One must remember that the above homomer/heteromer classification (i.e., the “=” and “≠” signs) refers to the domain fingerprints, not the sequence of the chains.

The improved EP algorithm was run twice on each assembly: with outlier detection (*r* = 3) and without it (*r* = 0). Visualization of the results via *EI*_0.3_ × *EI*_1.0_ maps (i.e., in the form of Figure 3b) is available in Supplemental File S7—Figures S27–S41, while the summary of the globularity metrics is given in Table 6 (*r* = 3) and Table 7 (*r* = 0). The values of those metrics for all individual assemblies are in Supplemental Files S8 (*r* = 3) and S9 (*r* = 0).

**Figure 6:**
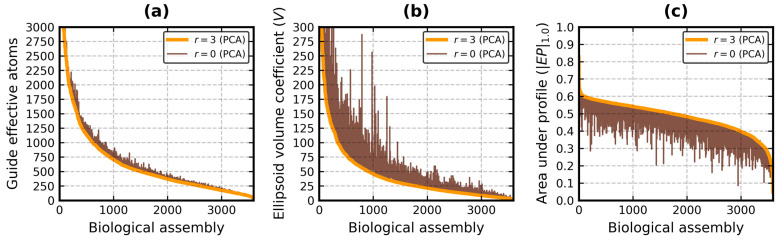
Comparison of selected globularity metrics returned by the improved EP algorithm (PCA) for the 3594 representatives of the biological assemblies from the PDB with outlier detection turned on (*r* = 3) and off (*r* = 0): (**a**) number of guides, (**b**) *V* coefficient and (**c**) *|EP|*_1.0_. The results are sorted in decreasing order of the value of the metrics for *r* = 3.

Figure 6 displays comparisons of three metrics of the EP algorithm: number of guide effective atoms, *V* and *|EP|*_1.0_. These charts look similar to the charts from Figure 5.

The number of common PDB codes between the structures containing the biological assemblies from this database and the structures containing the representatives of SCOP domain superfamilies was 854, which is 40.2% of 2124 and 23.8% of 3594, respectively.

Asymmetric biological assemblies constituted 50.3% of the database. In total, 65.5% of them were monomeric and 55.5% of those monomers had only one domain. The next 28.6% of the database was occupied by complexes exhibiting the C_2_ symmetry. A total of 84.1% of them had fingerprint-wise homomeric chains (i.e., groups C2o= and C2m=) and 62.1% of those homomers had one domain in their chains (i.e., group C2o=). Structures with dihedral symmetries were in the minority (12.2%) and only 27 molecules possessed either the tetrahedral, octahedral or icosahedral symmetries (i.e., the members of the TOI group). 60 chains comprised each of the four largest complexes in the database.

The EP algorithm employed on average 94% (*σ* = 4%) of the residues as guides for the bounding ellipsoid subroutine. Their numbers increased with the numbers of residues in the symmetric proteins, reaching 99% in TOI, 98% in D4+ and 97% in D3. This is understandable—the more regular the structure is, the less outliers are found in it by PCA.

The single-domain monomers (A1o) were the smallest proteins, showing an average value of the *V* coefficient at *r* = 3 equal to 8.6 (*σ* = 7.1), close to the *V* data in the last row of Table 4 (μ = 7.4, *σ* = 7.2). The next group in this sense was C2o= (μ = 21.5, *σ* = 23.6), comparable with the multi-domain monomers (A1m, μ = 23.4, *σ* = 15.7). Conversely, the largest asymmetric complexes (A4+) were the third group bound in the largest MVEEs (μ = 133.8, *σ* = 244.1) after TOI and D4+ (n.b., they were even the second largest at *r* = 0). With seven chains on average (*σ* = 6.7), they also exhibited many visually diverse conformations.

While the average values of *EI*_0.3_ and *EI*_1.0_ for all domain superfamily representatives together reached 0.5 at *r* = 3 (0.57 and 0.5 to be precise, in the zone of high globularity), only the single-domain monomers (A1o) attained a similar status (*EI*_0.3_ = 0.58, *EI*_1.0_ = 0.51). This corroborates the above observation of the closeness of these sets in terms of the *V* coefficient. The average ellipsoid index values of all biological assembly representatives were 0.51 and 0.44. This puts them as a whole in the *G* class zone, although just barely. The ellipsoid indexes in the A1m, A2 and C2o= groups were close to each other with their average *|EP|*_1.0_ values between 0.49 and 0.51 (*EI*_0.3_ > 0.5, *EI*_1.0_ ≈ 0.45). After all, they were mostly pairs of domains. Groups A3, C2m=, C2o≠, C3 and D2 had their average *EI*_0.3_ between 0.47 and 0.49 and average *EI*_1.0_ equal to 0.41 (*|EP|*_1.0_ ≈ 0.45). Index similarities were also discovered between A4+, C2m≠, C4+ and D3 groups. However, their average *EI*_1.0_ were below 0.4 (*|EP|*_1.0_ ≈ 0.4). Only groups D4+ and TOI were worse in this regard. Both had average *EI*_0.3_ below 0.4. TOI even dropped below 0.3, making it the only intrinsically non-globular subset here. Both groups also achieved low average values of *|EP|*_0.1_ (0.05 and 0.02). This strongly hints at the primary cause of their low ellipsoid indexes.

At *r* = 3, 61.7% of the biological assemblies was considered globular by the EP algorithm. In total, 74% of them belonged to groups A1o, A1m, A2 and C2o= (A1o alone constituted 36.9% of this 74%). This is not far away from the percent of the domain superfamily representatives in class *G* (69.8%). A total of 55% of all highly globular assemblies originated from group A1o. However, the total percent of members of class *H* was lower than among the domains, 23.5% of 3594, down from 61.5% of 2124. Conversely, 31.7% of the assemblies and 10% of the domains comprised class *S*. In total, 44.8% of this class originated from groups A1m, C2o=, C2m= and D2 (over 100 structures in each). On the other hand, the fractions of the non-globular molecules were comparable: 6.6% of the assemblies versus 4.1% of the domains. Groups A1o, A2, A3 and C2o≠ had less than 11 members in class *N* (group C2o≠ had only two, but also two in class *H*). No row in Table 4 has less numbers for class *G* than for the two other main classes combined. Here, only groups A1o, A1m, A2, C2o= and C3 exhibited this property. As expected after the previous paragraph, groups D4+ and TOI were their opposites, with a total of five globular structures in them. That lone “green” molecule in group TOI (Figure S41 in Supplemental File S7) is the tetrahedral UbiX complex (12 × c.34.1.0 domains) with PDB code 4ZAV [67]. Unlike the other members of its group, it did not feature an overly large void in its center (only a relatively small one, but detectable due to *|EP|*_0.1_ = 0.11), which allowed it to barely pass into class *G* (*EI*_0.3_ = 0.5, *EI*_1.0_ = 0.452). It was also not assigned to class *U*, whereas 77.8% of TOI was. The percent of unusual results increased to 7.8% from 5.1% for the domains. In total, 62.1% of those 7.8% was in groups C4+, D2, D3, D4+ and TOI. With the sole exception of D2, more than 25% of their members were in class *U*. Lastly, 6% of the domains were considered elongated (class *E*), but this property carried only to 1.1% of the assemblies, 39 to be exact. 20 of them were in groups A1o and A1m, followed by four in A2 and C2m=.

The results at *r* = 0 demonstrate how important the outlier detection subroutine is for the measurement of globularity and how it supports the entire workflow even when no significant outliers are presents. Compared with results at *r* = 3, the *V* coefficient increased on average by 16.5 (*σ* = 29.1), followed by an average drop of *EI*_0.3_ by 0.03 (*σ* = 0.05) and of *EI*_1.0_ by 0.06 (*σ* = 0.04). The average values of *EI*_0.3_ and *EI_1.0_* for the entire set of assemblies stopped at 0.47 (*σ* = 0.11) and 0.37 (*σ* = 0.09), respectively. This places them in the *S* class zone (n.b., the domain set stayed in class *G*—Table 5). Groups A4+ and C2m≠ joined group TOI in being primarily located in class *N* (*EI*_0.3_ ≈ 0.38, *EI*_1.0_ ≈ 0.28). The number of assemblies in class *G* changed from 2218 to 1704 (1688 stayed there since *r* = 3). For the other classes these numbers were as follows: 843 to 265 (265) in class *H*, 1138 to 1149 (743) in class *S*, 238 to 741 (236) in class *N*, 282 to 210 (195) in class *U* and 39 to 25 (23) in class *E*.

With an observed |*EP*|_1.0_ difference higher than or equal to 15%, 21 assemblies dropped from class *G* to class *S*, 55 dropped from class G to class *N* and 31 dropped from class *S* to class *N*. Again, 55 is the number of molecules suspected of having significantly outlying fragments. 46 of them were in groups A1o, A1m, A2, C2o= and C2m=. Group A1o witnessed 14 of the 21 switches from class *G* to class *S*. One of the examples is the catalytic core and C-terminal domain of HIV-1 Integrase with PDB code 1EX4 [68] and domain fingerprint (c.55.3.2,b.34.7.1)*2. Its C-terminal domains are outlying at *r* = 3. Like in 1DIV, the helices that link them to the core are also isolated at *r* = ∞.

## 4. Conclusions

This paper reports the findings of the continuation of our initial research in the area of the measurement of globularity of proteins via voxel-based atom representation and the approximation of their shape via minimum volume enclosing ellipsoids [22]. The tool used for this measurement is called the ellipsoid profile algorithm (EP) [22].

The EP algorithm was recalled in Section 2.3 to further establish its main concepts in a way that should be convenient for programmers. In fact, it can be readily implemented using functions from the popular computational packages such as NumPy and SciPy. The brief code of its only “external” subroutine (MVEE) is also available online [69,70]. This description of the EP algorithm is supported by additional explanations and a minor addition of the new labels for the classes of globularity (*N*, *S*, *G*, *H*, *U*, *E*).

In Section 3.1, we presented a modified version of the EP algorithm enhanced with an improved subroutine for the detection of significant structural outliers (e.g., portions of the chain extended into solvent that can negatively impact the measurement of globularity). This enhancement is based on principal component analysis (PCA) in tandem with the confidence ellipsoid method. We recently introduced this idea to the calculation pipeline of the fuzzy oil drop model (FOD) [26]. It was applied to the EP algorithm with a few tweaks needed for its context. It replaces the current subroutine based on medians of kernel density estimation (KDE) as the default outlier detection workflow. PCA quickly and precisely aligns the input set of effective atoms with the axes of the coordinate system, while the subsequent confidence ellipsoid splits this set between the outliers and guides (i.e., the elements of the “main body” of the protein). Size of the confidence ellipsoid (i.e., the strength of the outlier detection) is controlled by the confidence level parameter *P*. Its suggested default value is 0.9. It seems to be stable and robust against the various kinds of structures (with and without outliers) when used along *r* = 3, which is the parameter controlling the maximum number of detection rounds. When *r* = 0 the entire detection is bypassed. Analysis and comparison of globularity metrics at *r* > 0 and *r* = 0 informs about the possibility of structural issues in the input structure, such as the previously mentioned outlier fragments, elongation or central void, such as that in 3BPD:(A–G).

One may be tempted to run these two techniques in succession, first PCA then KDE, on the guides returned by PCA. However, to not overdo it, *m*, the number of kernel density medians, should be increased from 3 to 4, which eventually does not change much in terms of the globularity metrics but burdens the CPU with an extra workload. To achieve PCA’s anti-outlier properties by KDE in tandem with a 1-*d* confidence ellipsoid, *P* would need to be lowered to ≈0.5 (and *r* set to 1), which causes too much of the molecule to be carved into from all directions. Owing to two parameters with sensible defaults (one being real-valued), the new solution can be tweaked for various experiments with finer granularity. However, we do not completely abandon the KDE-based approach. It can be useful for some proteins due to its ability to scrutinize their shape from a different perspective. Our web server at http://fod.cm-uj.krakow.pl retains it as an option.

In Section 3.2 we applied the improved EP algorithm to the six example proteins and in Section 3.3 we ran it on the 2124 representatives of SCOP domain superfamilies from the modified ASTRAL compendium, comparing its outcome to the previous (i.e., KDE-based) results from ref. [22]. It brought a fresh look on the globularity of the majority of structural types of protein domains and allowed us to reach the above conclusions.

In Section 3.4 we expanded the survey of the landscape of globularity from protein domains (i.e., at the tertiary structure level) to manually assigned biological assemblies (i.e., at the quaternary structure level) that were extracted from models deposited in the PDB. This way, we measured the status of the functional forms of the molecules that can be found in vivo. The EP algorithm was applied to the 3594 representatives of biological assemblies with various complex sizes and symmetries, ranging from monomers to sizable complexes such as Urease. The structures were divided between their symmetry classes (cyclic, dihedral, etc.) for detailed analysis. The selection, clustering and validation of the assembly information was performed with the help of the 3DComplex database and custom SCOP domain fingerprints. The results are in line with the expectations, positively validating the EP algorithm. They complement the data obtained for the domains.

We believe that the EP algorithm is a useful addition to structural biologists’ and bioinformaticians’ toolboxes, especially for those interested in checking whether the given protein is globular or not. It can be applied to any part of the molecule and is aware of the unoccupied space within its bounding ellipsoid (MVEE). The use of effective atoms with the outlier detection allows the EP algorithm to produce tight ellipsoidal representations of the proteins that are less affected by local features of the molecular surface. Research aimed at its further improvements and applications will continue.

## Figures and Tables

**Table 1:** The 6 proteins used to illustrate the various inputs and outputs of the EP algorithm.

PDB Code	Molecule	Source Organism	Chain Length	SCOP Domain(s)	Quaternary Structure	Resolution	Ref.
1IS9	Endoglucanase A	*Clostridium thermocellum*	363 aa!	a.102.1.2	monomer	1.03 Å	[41]
3BPD	Uncharacterized Protein	*Archaeoglobus fulgidus*	100 aa!	d.58.61.1	homo-7-mer	2.80 Å	[42]
4B0H	dUTPase YncF	*Bacillus subtilis*	144 aa!	b.85.4.0	homo-3-mer	1.18 Å	[43]
1IIE	HLA-DR Invariant Chain	*Homo sapiens*	75 aa	a.109.1.1	homo-3-mer	NMR (1/20)	[44]
2KKW	Alpha Synuclein	*Homo sapiens*	140 aa	h.7.1.1	monomer	NMR (1/34)	[45]
1DIV	Ribosomal Protein L9	*Bacillus stearothermophilus*	149 aa	d.99.1.1 d.100.1.1	homo-2-mer	2.60 Å	[46]

Exclamation mark in the chain length column informs that the number of residues available in the PDB structure is lower than the length of the sequence listed in the SEQRES records. Quaternary structure column presents the stoichiometry of the biological assembly (all 4 complexes have cyclic symmetry). “1/*n*” in the resolution column refers to the fact that only NMR conformer 1 out of the total of n conformers deposited in the PDB file was used in this study.

**Table 2:** Globularity metrics of proteins from Table 1 with outlier detection (*r* = 3).

PDB Code	Selected Chains	Effective Atoms		Bounding Ellipsoid					Ellipsoid Index		Ellipsoid Profile		Globularity Classes
		All	Guide	*r*_*x*_	*r*_*y*_	*r*_*z*_	*V*	*T*	*EI*_0.3_	*EI*_1.0_	*|EP|*_0.1_	*|EP|*_1.0_	
1IS9	A	358	354	27	24	23	14.9	0.57	0.589	0.561	0.541	0.590	*G* and *H*
3BPD	A–G	638	612	39	39	23	35.0	0.63	0.207	0.344	0.000	0.275	*N* and *U*
4B0H	B	143	111	24	19	13	5.9	0.75	0.597	0.498	0.526	0.560	*G*
1IIE	A–C	225	198	22	22	18	8.7	0.55	0.713	0.592	0.781	0.662	*G* and *H*
2KKW	A	140	119	62	23	19	27.1	1.48	0.086	0.084	0.107	0.087	*N*
1DIV	A+B	298	244	45	35	22	34.6	0.79	0.300	0.198	0.493	0.261	*N*

Selected chains column denotes chains from the structure with given PDB code that were passed as the input to the EP algorithm. All is the number of all effective atoms, equal to the number of input residues, whereas guide is the number of guide effective atoms. *r*_*x*_, *r*_*y*_, *r*_*z*_ are the lengths of the radii of the bounding ellipsoid. Underlined values and class labels promote the globular status.

**Table 3:** Globularity metrics of proteins from Table 1 without outlier detection (*r* = 0).

PDB Code	Selected Chains	Effective Atoms		Bounding Ellipsoid					Ellipsoid Index		Ellipsoid Profile		Globularity Classes
		All	Guide	*r*_*x*_	*r*_*y*_	*r*_*z*_	*V*	*T*	*EI*_0.3_	*EI*_1.0_	*|EP|*_0.1_	*|EP|*_1.0_	
1IS9	A	358	358	29	25	22	15.9	0.62	0.593	0.541	0.587	0.581	*G* and *H*
3BPD	A–G	638	638	40	39	29	45.2	0.59	0.176	0.297	0.002	0.237	*N* and *U*
4B0H	B	143	143	34	26	21	18.6	0.72	0.214	0.197	0.177	0.210	*N*!
1IIE	A–C	225	225	44	44	22	42.6	0.67	0.387	0.189	0.413	0.295	*N*!
2KKW	A	140	140	60	42	22	55.4	0.94	0.031	0.047	0.004	0.039	*N* and *U*!
1DIV	A+B	298	298	50	50	23	57.5	0.69	0.227	0.149	0.462	0.200	*N*

Meaning of the symbols is retained from Table 2. At *r* = 0 all effective atoms are the guides. Exclamation mark in the globularity classes column denotes globularity status change versus Table 2. Underlined values and class labels promote the globular status.

**Table 4:** Statistical summary of the results of the application of the improved EP algorithm to the 2124 SCOP domain superfamily representatives with outlier detection (*r* = 3).

Domains			Effective		Guides		*V*		*EI*_0.3_		*EI*_1.0_		*|EP|*_0.1_		*|EP|*_1.0_		Globularity Classes					
cl	cf	sf	μ	*σ*	μ	*σ*	μ	*σ*	μ	*σ*	μ	*σ*	μ	*σ*	μ	*σ*	*N*	*S*	*G*	*H*	*U*	*E*
*a*	290	519	134	90	93%	5%	6.3	5.3	0.55	0.11	0.49	0.09	0.52	0.17	0.53	0.10	23	73	423	309	43	21
*b*	179	374	162	101	93%	5%	7.3	5.7	0.58	0.07	0.51	0.06	0.54	0.14	0.56	0.06	9	20	345	265	15	2
*c*	147	246	233	105	95%	4%	10.6	5.9	0.58	0.05	0.51	0.05	0.59	0.10	0.55	0.05	2	10	234	168	5	0
*d*	395	577	141	78	93%	5%	6.4	4.5	0.57	0.07	0.50	0.07	0.56	0.13	0.55	0.07	18	34	525	376	10	5
*e*	73	73	360	204	92%	5%	20.3	16.7	0.51	0.10	0.44	0.08	0.51	0.15	0.48	0.09	5	16	52	17	3	0
*f*	69	130	176	144	88%	6%	9.2	8.9	0.54	0.18	0.46	0.14	0.53	0.24	0.51	0.16	16	33	81	42	14	40
*g*	98	139	61	31	93%	5%	2.4	2.0	0.60	0.12	0.53	0.11	0.62	0.20	0.57	0.11	4	15	120	98	10	3
*h*	6	66	105	83	89%	7%	7.2	14.1	0.56	0.23	0.48	0.17	0.53	0.28	0.53	0.20	10	12	44	31	8	56
all	1257	2124	157	113	93%	5%	7.4	7.2	0.57	0.10	0.50	0.09	0.55	0.16	0.54	0.09	87	213	1824	1306	108	127

cl—SCOP domain class; cf—number of unique common folds in this class; sf—number of unique superfamilies in this class; *N*, *S*, *G*, *H*, *U*, *E*—globularity classes; μ—average; *σ*—standard deviation.

**Table 5:** Statistical summary of the results of the application of the improved EP algorithm to the 2124 SCOP domain superfamily representatives without outlier detection (*r* = 0).

Domains			Effective		Guides	*V*		*EI*_0.3_		*EI*_1.0_		*|EP|*_0.1_		*|EP|*_1.0_		Globularity Classes					
cl	cf	sf	μ	*σ*	100%	μ	*σ*	μ	*σ*	μ	*σ*	μ	*σ*	μ	*σ*	*N*	*S*	*G*	*H*	*U*	*E*
*a*	290	519	134	90	100%	9.5	8.7	0.51	0.13	0.41	0.11	0.51	0.19	0.47	0.12	78	106	335	117	37	13
*b*	179	374	162	101	100%	11.1	10.8	0.54	0.10	0.43	0.09	0.54	0.15	0.50	0.10	43	31	300	76	8	1
*c*	147	246	233	105	100%	14.4	8.9	0.55	0.08	0.44	0.07	0.57	0.12	0.51	0.07	10	39	197	56	2	0
*d*	395	577	141	78	100%	9.5	9.8	0.53	0.11	0.43	0.09	0.55	0.15	0.49	0.10	71	64	442	130	9	2
*e*	73	73	360	204	100%	31.4	29.4	0.46	0.11	0.36	0.09	0.48	0.15	0.42	0.10	21	21	31	0	3	0
*f*	69	130	176	144	100%	15.6	15.2	0.43	0.18	0.35	0.13	0.43	0.25	0.40	0.15	46	35	49	13	21	30
*g*	98	139	61	31	100%	3.7	3.4	0.55	0.14	0.46	0.12	0.59	0.20	0.51	0.13	18	19	102	57	5	0
*h*	6	66	105	83	100%	12.3	18.8	0.44	0.23	0.37	0.18	0.42	0.30	0.41	0.21	28	11	27	17	19	46
all	1257	2124	157	113	100%	11.2	12.3	0.52	0.13	0.42	0.11	0.53	0.18	0.48	0.12	315	326	1483	466	104	92

Meaning of the symbols is retained from Table 4. At *r* = 0 all effective atoms are the guides.

**Table 6:** Statistical summary of the results of the application of the improved EP algorithm to the 3594 representatives of the biological assemblies from the PDB with outlier detection (*r* = 3).

Assemblies		Effective		Guides		*V*		*EI*_0.3_		*EI*_1.0_		*|EP|*_0.1_		*|EP|*_1.0_		Globularity Classes					
Group	Count	μ	*σ*	μ	*σ*	μ	*σ*	μ	*σ*	μ	*σ*	μ	*σ*	μ	*σ*	*N*	*S*	*G*	*H*	*U*	*E*
A1o	657	187	119	95%	4%	8.6	7.1	0.58	0.06	0.51	0.06	0.56	0.13	0.56	0.06	8	43	606	464	17	7
A1m	527	416	223	93%	4%	23.4	15.7	0.52	0.08	0.45	0.07	0.51	0.15	0.49	0.07	17	132	378	101	17	13
A2	352	445	227	93%	4%	25.7	17.2	0.52	0.07	0.44	0.06	0.53	0.13	0.49	0.07	10	92	250	51	4	4
A3	169	607	285	92%	3%	36.9	21.5	0.49	0.06	0.41	0.05	0.48	0.13	0.46	0.05	5	95	69	3	2	2
A4+	103	1589	1597	92%	4%	133.8	244.1	0.42	0.10	0.35	0.07	0.37	0.19	0.39	0.09	27	54	22	0	10	0
C2o=	537	392	324	94%	4%	21.5	23.6	0.53	0.08	0.46	0.06	0.46	0.15	0.51	0.07	14	115	408	164	19	2
C2m=	328	784	401	93%	4%	48.2	28.2	0.48	0.07	0.41	0.06	0.43	0.15	0.45	0.07	19	148	161	10	12	4
C2o≠	83	807	408	92%	4%	48.0	25.0	0.49	0.07	0.41	0.05	0.42	0.16	0.46	0.06	2	40	41	2	4	0
C2m≠	81	1449	1001	91%	5%	102.2	79.5	0.43	0.08	0.36	0.07	0.36	0.18	0.41	0.08	15	47	19	2	5	2
C3	184	799	586	94%	5%	50.4	42.8	0.49	0.09	0.41	0.07	0.34	0.17	0.46	0.08	17	68	99	18	17	3
C4+	109	1256	918	96%	4%	87.7	78.2	0.40	0.12	0.39	0.07	0.16	0.16	0.40	0.09	27	55	27	7	48	0
D2	224	1172	736	95%	4%	75.5	58.3	0.47	0.08	0.41	0.06	0.28	0.16	0.45	0.07	12	115	97	12	29	1
D3	134	1772	1001	97%	3%	121.9	80.9	0.43	0.10	0.39	0.06	0.16	0.13	0.41	0.08	21	77	36	9	34	1
D4+	79	2323	1629	98%	2%	165.6	140.3	0.35	0.12	0.37	0.06	0.05	0.07	0.36	0.08	26	49	4	0	43	0
TOI	27	5080	3546	99%	1%	378.9	331.5	0.23	0.16	0.33	0.08	0.02	0.05	0.28	0.11	18	8	1	0	21	0
all	3594	702	895	94%	4%	45.1	79.6	0.51	0.10	0.44	0.08	0.44	0.19	0.48	0.09	238	1138	2218	843	282	39

Meaning of the symbols is retained from Table 4. Count is the number of assemblies in given group.

**Table 7:** Statistical summary of the results of the application of the improved EP algorithm to the 3594 representatives of the biological assemblies from the PDB without outlier detection (*r* = 0).

Assemblies		Effective		Guides	*V*		*EI*_0.3_		*EI*_1.0_		*|EP|*_0.1_		*|EP|*_1.0_		Globularity Classes					
Group	Count	μ	*σ*	100%	μ	*σ*	μ	*σ*	μ	*σ*	μ	*σ*	μ	*σ*	*N*	*S*	*G*	*H*	*U*	*E*
A1o	657	187	119	100%	11.8	10.3	0.55	0.08	0.45	0.08	0.56	0.13	0.51	0.08	29	85	543	184	10	3
A1m	527	416	223	100%	34.4	25.7	0.48	0.10	0.37	0.08	0.48	0.16	0.44	0.09	84	169	274	15	10	10
A2	352	445	227	100%	39.0	28.9	0.48	0.09	0.36	0.07	0.51	0.14	0.43	0.08	77	96	179	4	5	1
A3	169	607	285	100%	56.4	38.2	0.44	0.07	0.33	0.06	0.47	0.14	0.39	0.06	41	93	35	0	1	0
A4+	103	1589	1597	100%	199.0	271.3	0.37	0.11	0.28	0.07	0.35	0.18	0.33	0.09	52	44	7	0	6	0
C2o=	537	392	324	100%	30.5	34.4	0.50	0.10	0.40	0.08	0.45	0.15	0.46	0.09	75	127	335	43	17	3
C2m=	328	784	401	100%	70.8	42.9	0.44	0.09	0.34	0.07	0.41	0.15	0.40	0.07	93	148	87	2	8	4
C2o≠	83	807	408	100%	71.9	39.5	0.44	0.08	0.34	0.06	0.43	0.16	0.40	0.07	20	41	22	0	3	0
C2m≠	81	1449	1001	100%	170.8	146.4	0.39	0.10	0.28	0.08	0.35	0.18	0.34	0.09	49	20	12	1	1	1
C3	184	799	586	100%	71.1	64.8	0.46	0.10	0.36	0.08	0.34	0.17	0.41	0.09	49	60	75	6	14	3
C4+	109	1256	918	100%	107.7	93.5	0.38	0.12	0.35	0.08	0.16	0.16	0.36	0.10	42	47	20	4	39	0
D2	224	1172	736	100%	100.1	78.7	0.46	0.08	0.36	0.07	0.29	0.16	0.42	0.07	51	95	78	2	12	0
D3	134	1772	1001	100%	148.4	103.7	0.43	0.10	0.36	0.07	0.18	0.14	0.40	0.08	32	70	32	4	24	0
D4+	79	2323	1629	100%	186.2	156.3	0.35	0.12	0.36	0.07	0.06	0.08	0.35	0.08	31	44	4	0	41	0
TOI	27	5080	3546	100%	390.6	329.4	0.23	0.16	0.33	0.08	0.03	0.05	0.28	0.11	16	10	1	0	19	0
all	3594	702	895	100%	61.6	95.0	0.47	0.11	0.37	0.09	0.43	0.19	0.43	0.10	741	1149	1704	265	210	25

Meaning of the symbols is retained from Table 6. At *r* = 0 all effective atoms are the guides.

## Data Availability

Online access to the ellipsoid profile algorithm, the fuzzy oil drop algorithm and related bioinformatics tools is possible at the http://fod.cm-uj.krakow.pl web server.

## Supplementary Materials

The following supporting information can be downloaded at: https://www.mdpi.com/article/10.3390/biom13020385/s1. Supplemental File S1 (Figures S1–S6)—cartoon and surface style renders of the structures from Table 1; Supplemental File S2 (Figures S7–S12)—visualization of the bounding of the structures from Table 2 in the improved EP algorithm; Supplemental File S3 (Figures S13–S18)—visualization of voxelization of the structures from Table 2 in the improved EP algorithm; Supplemental File S4 (Figures S19–S26)—ellipsoid index maps of the representatives of the SCOP domain superfamilies calculated by the improved EP algorithm (size of the markers gauges the number of residues); Supplemental File S5—globularity metrics of the representatives of the SCOP domain superfamilies calculated using the improved EP algorithm with outlier detection; Supplemental File S6—like Supplemental File S5 but without outlier detection; Supplemental File S7 (Figures S27–S41)—like Supplemental File S4 but for the representatives of the biological assemblies from the PDB; Supplemental File S8—like Supplemental File S5 but for the representatives of the biological assemblies from the PDB; Supplemental File S9—like Supplemental File S8 but without outlier detection.
