# Supplementary material for: Improved Assessment of Globularity of Protein Structures and the Ellipsoid Profile of the Biological Assemblies from the PDB
Source: Biomolecules. 2023 Feb 17;13(2):385. doi: 10.3390/biom13020385 (PMC9953691; doi:10.3390/biom13020385)

Figure S13. Endoglucanase A – 1IS9:A

(a) $r = 3$ **(b)** $r = 0$

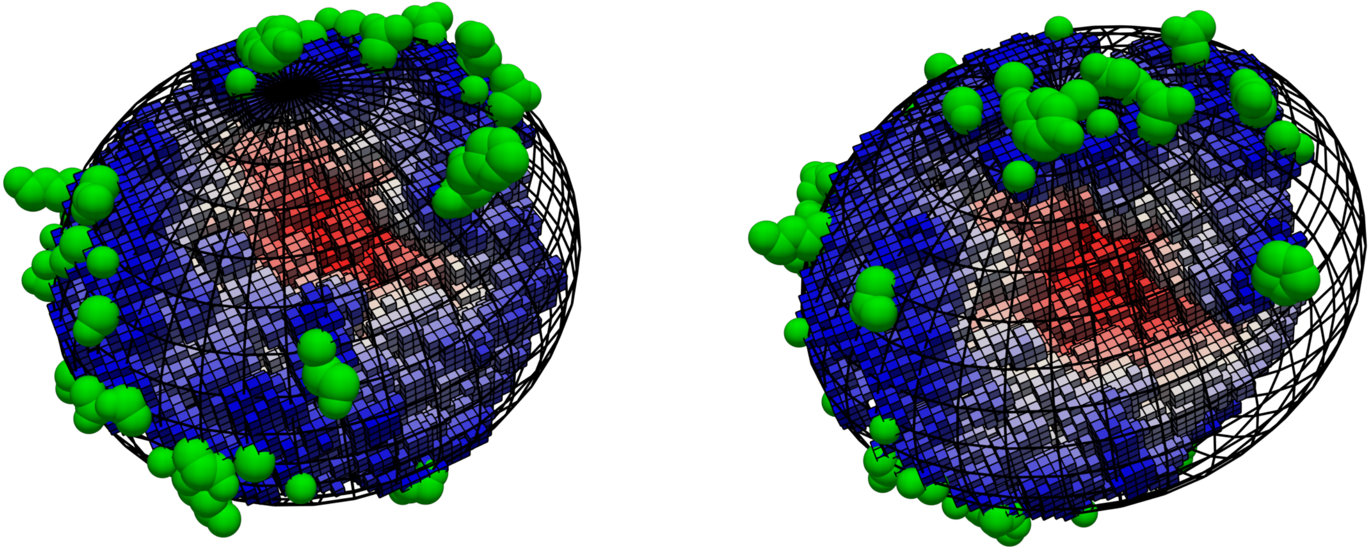

Figure S14. Uncharacterized Protein – 3BPD:(A–G)

(a) $r = 3$ **(b)** $r = 0$

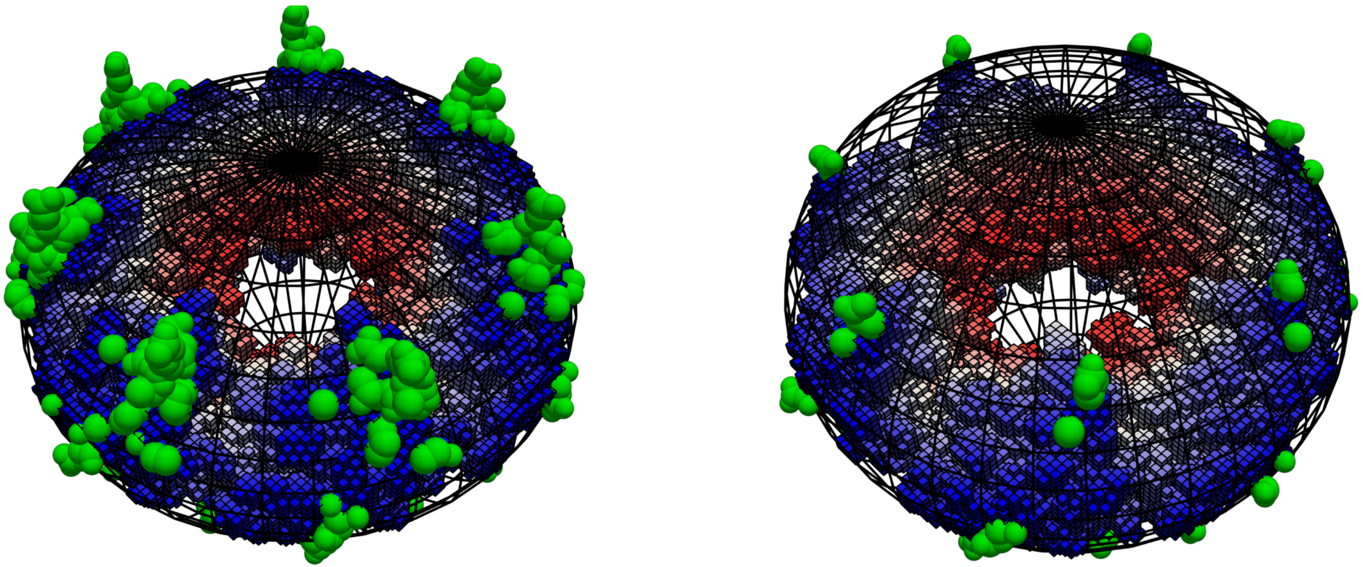

Figure S15. dUTPase YncF – 4B0H:B

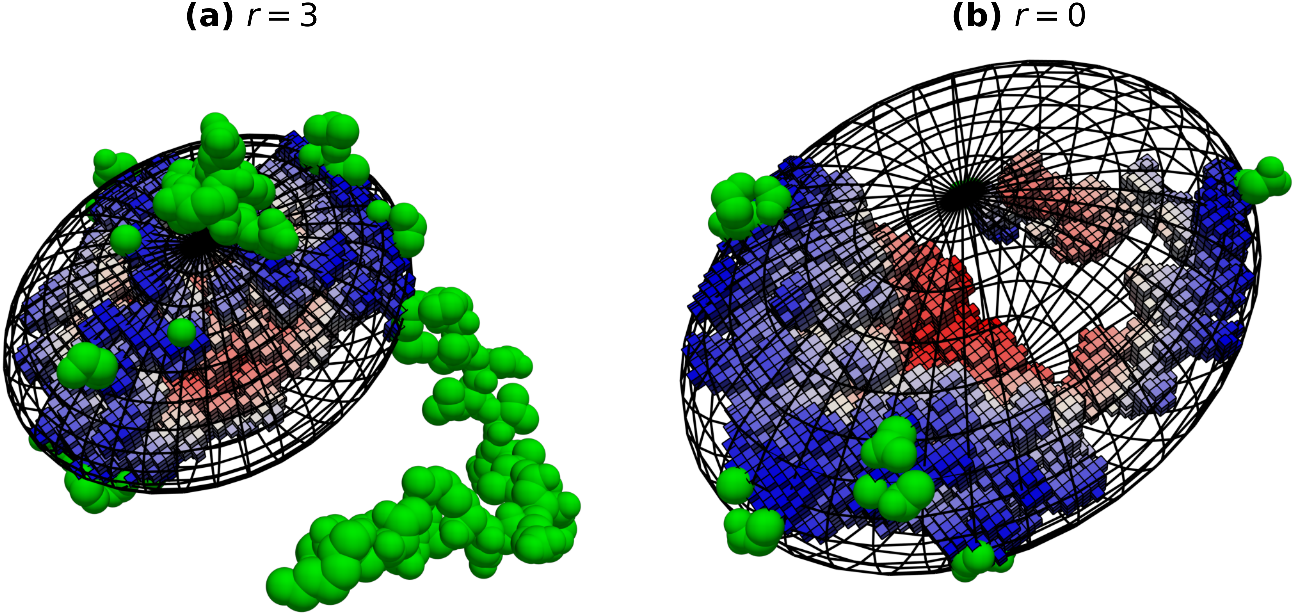

Figure S16. HLA-DR Invariant Chain – 1IIE:(A–C)

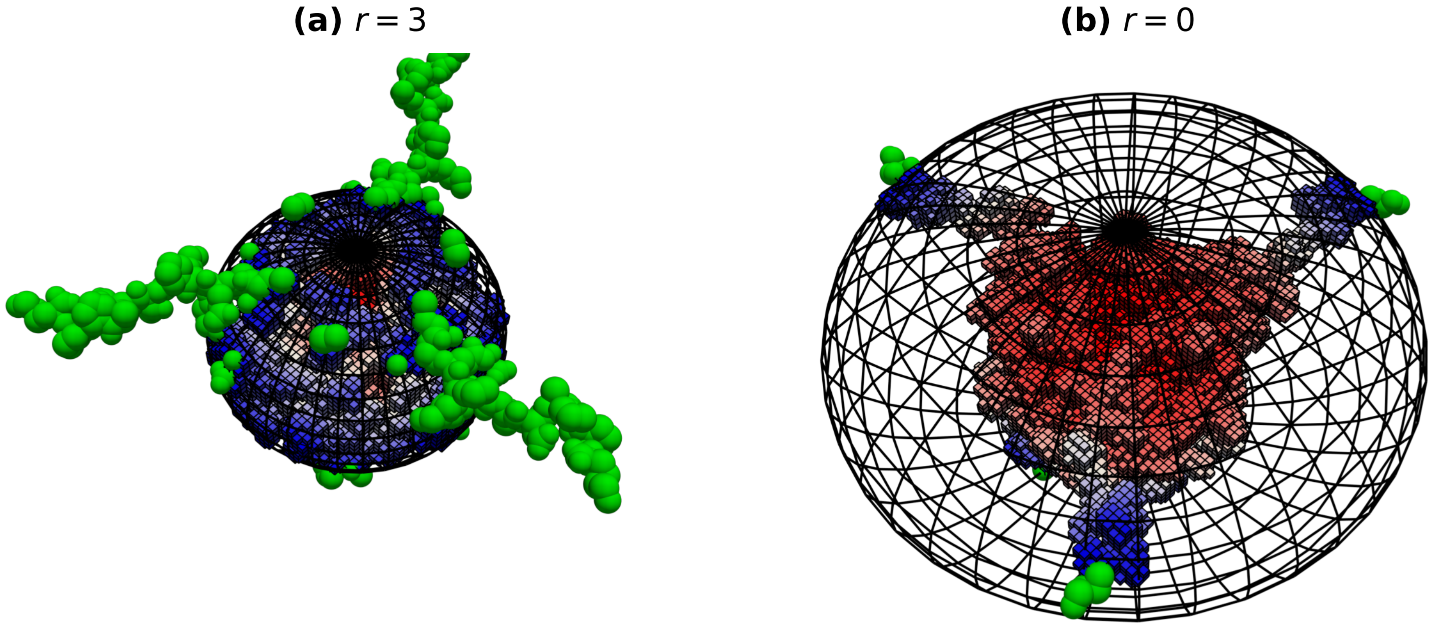

Figure S17. Alpha Synuclein – 2KKW:A

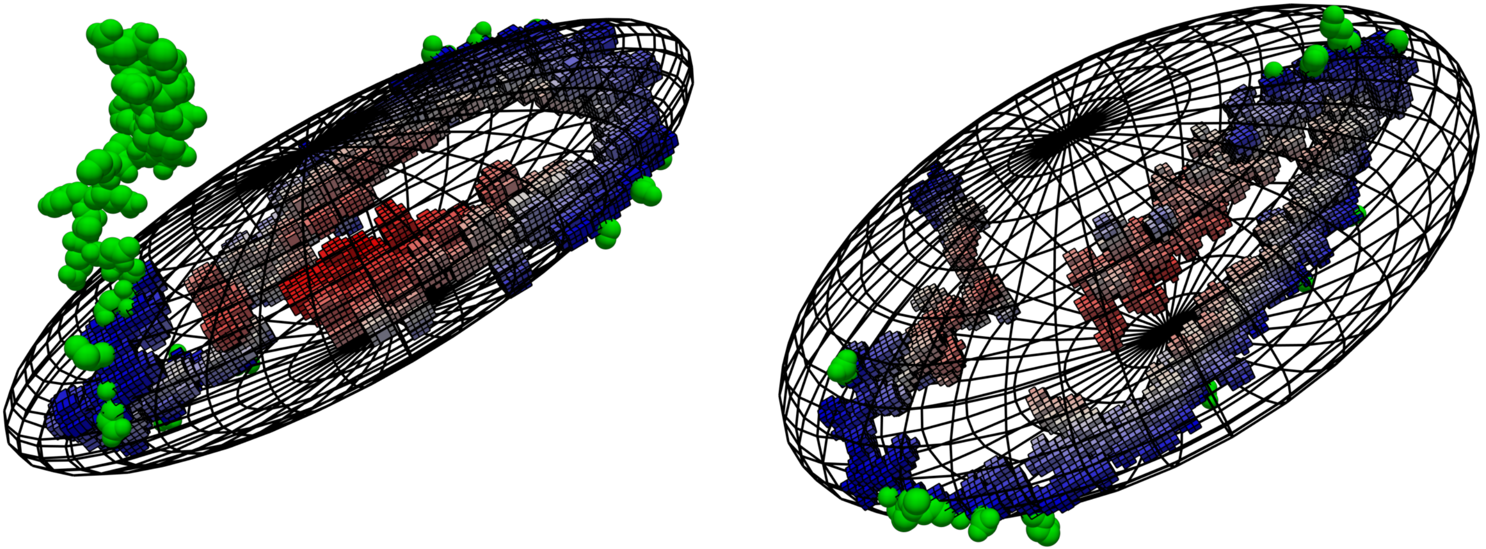

(a) $r = 3$ **(b)** $r = 0$

Figure S18. Ribosomal Protein L9 – 1DIV:(A+B)

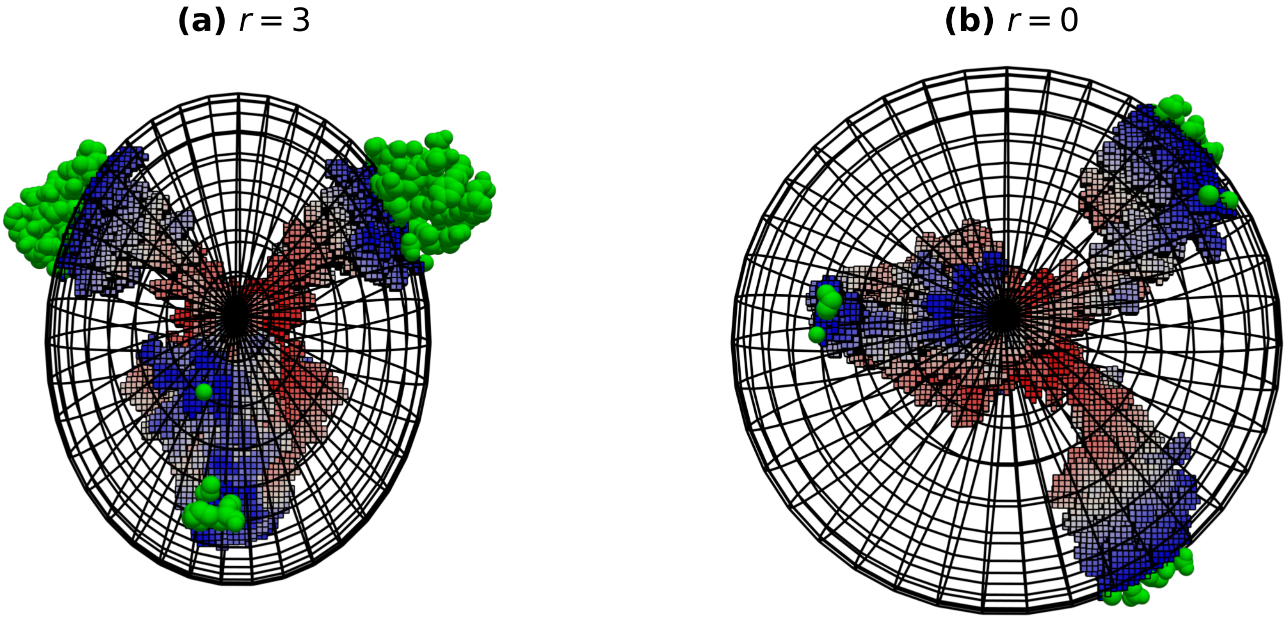

Supplement: Supplementary file 1 [file biomolecules-13-00385-s001.zip › s3.pdf]
