# Supplementary material for: Improved Assessment of Globularity of Protein Structures and the Ellipsoid Profile of the Biological Assemblies from the PDB
Source: Biomolecules. 2023 Feb 17;13(2):385. doi: 10.3390/biom13020385 (PMC9953691; doi:10.3390/biom13020385)

Figure S19. SCOP class a – all alpha proteins

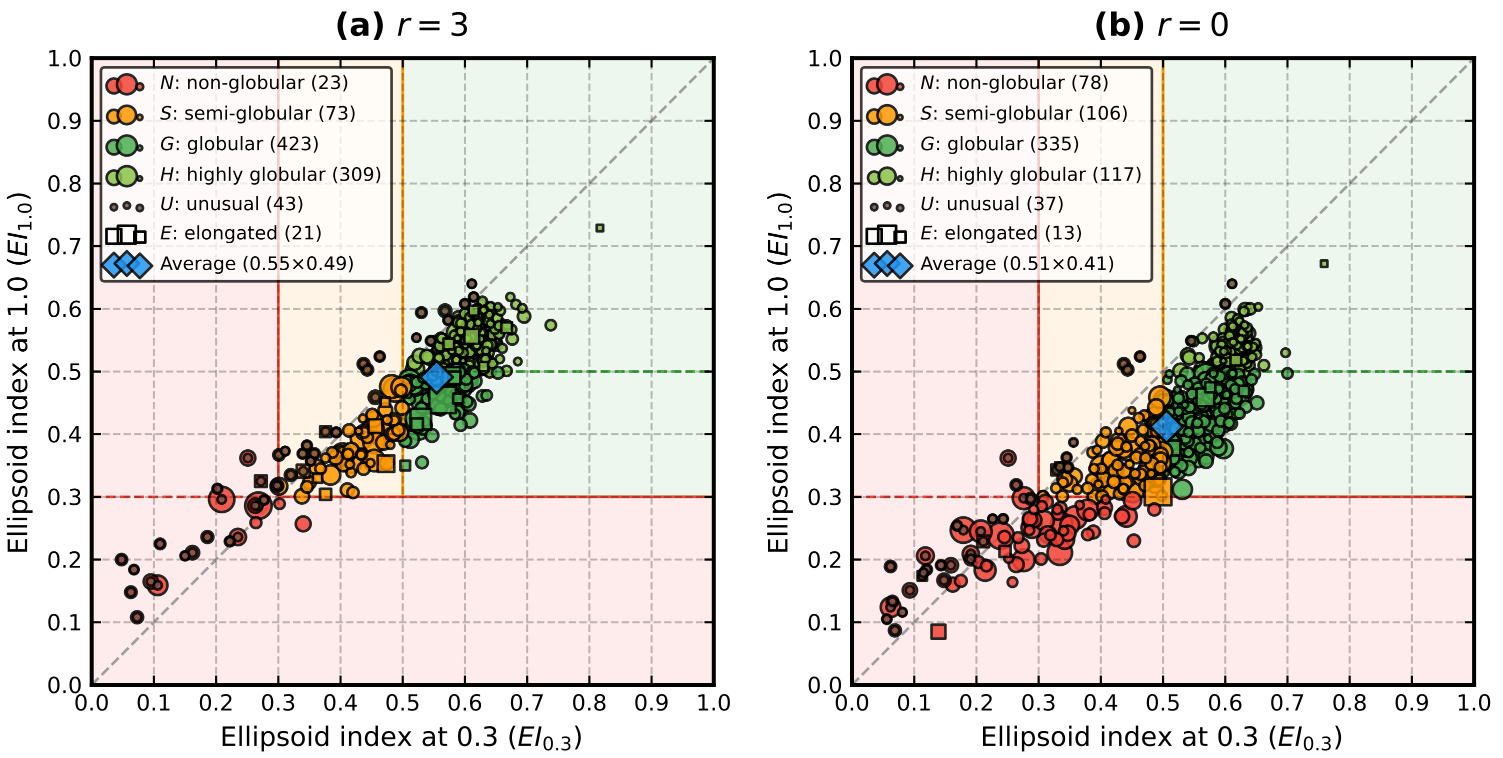

Figure S20. SCOP class b – all beta proteins

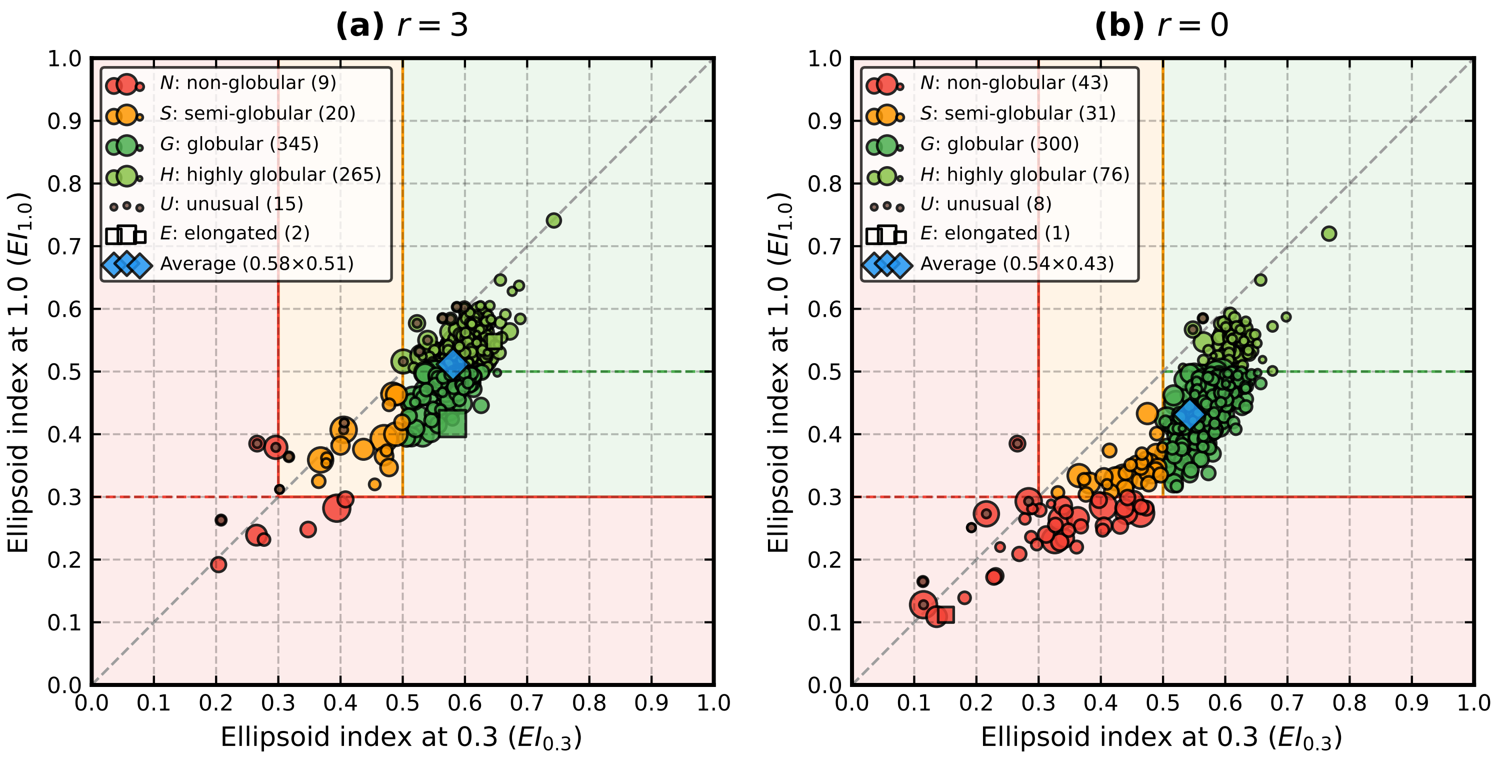

Figure S21. SCOP class c – alpha and beta proteins (a/b)

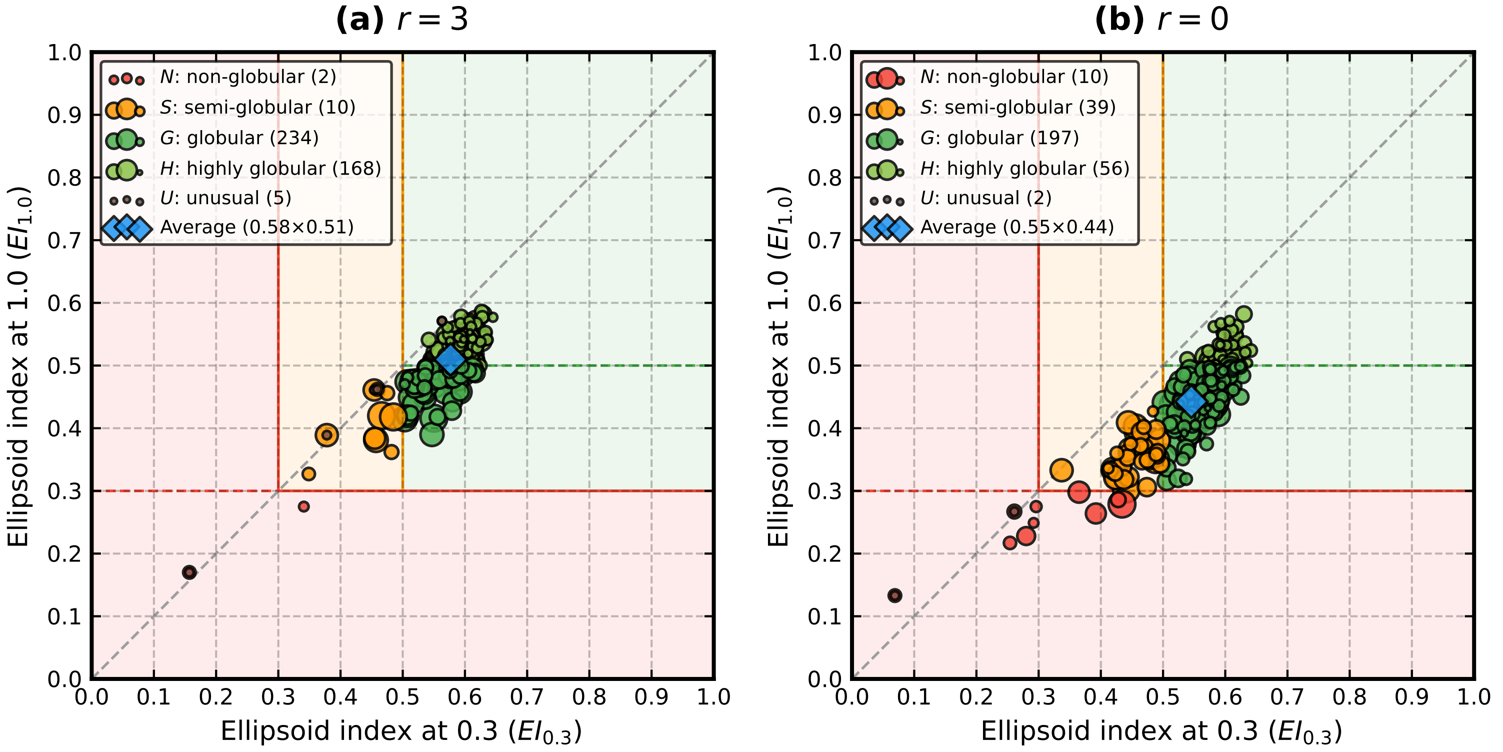

Figure S22. SCOP class d – alpha and beta proteins (a+b)

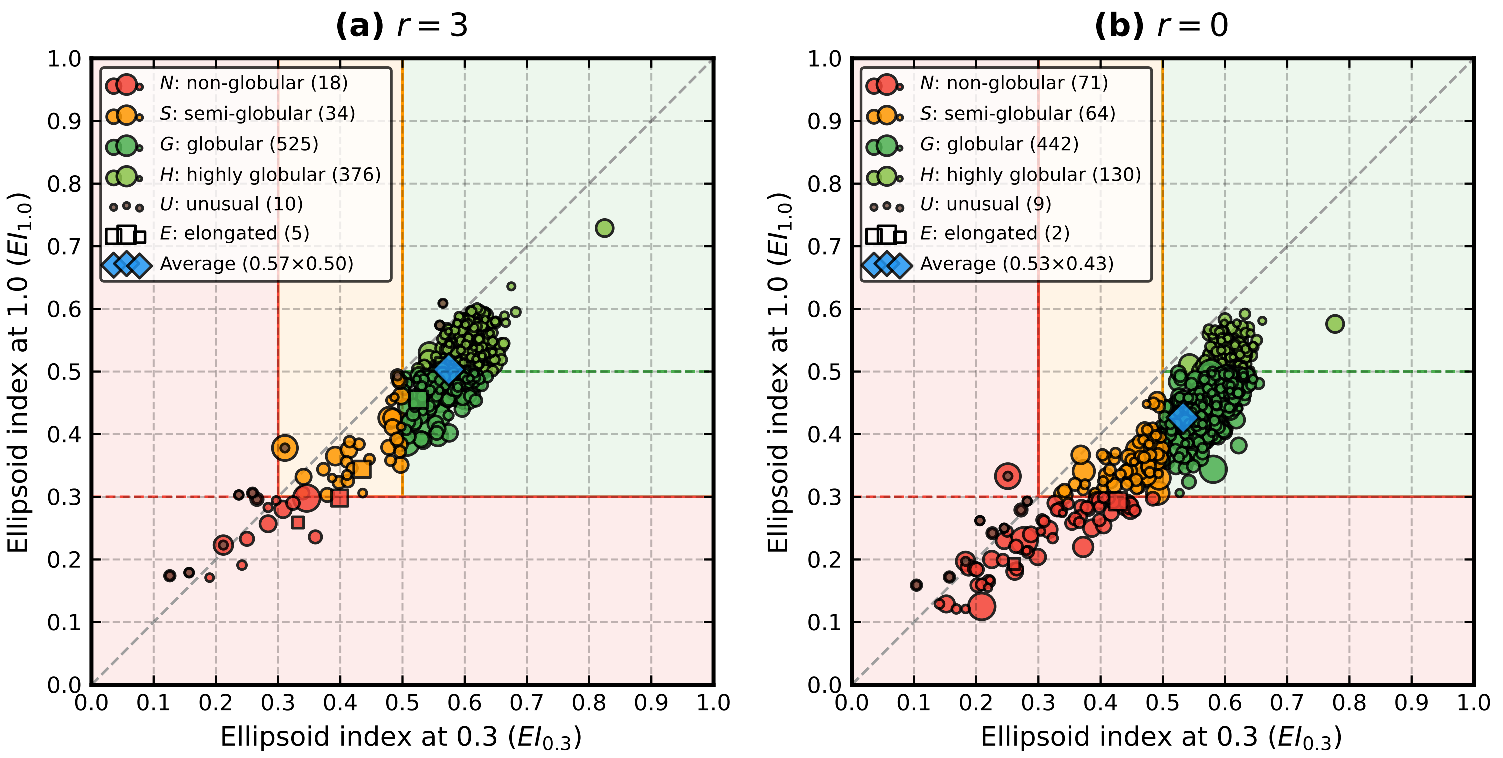

Figure S23. SCOP class e – multi-domain proteins

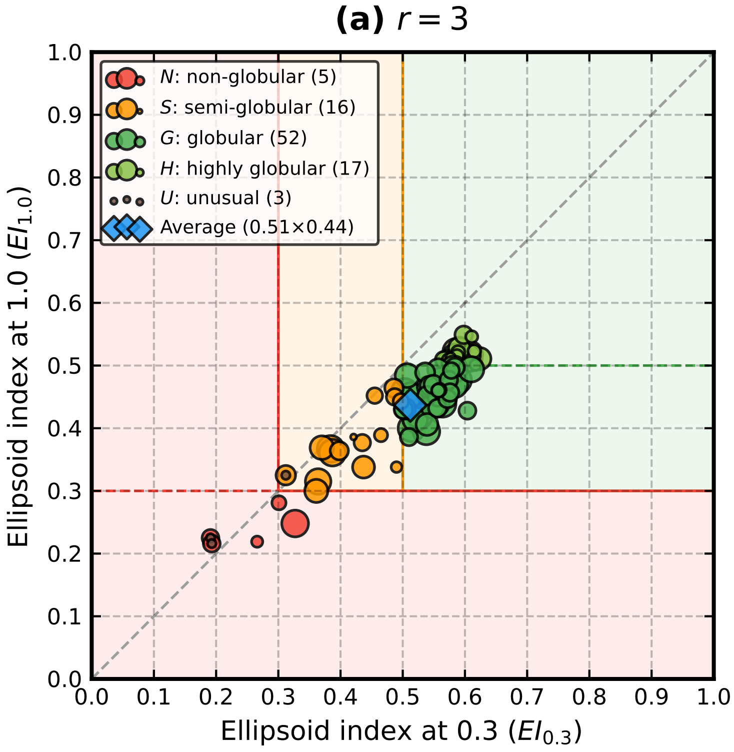

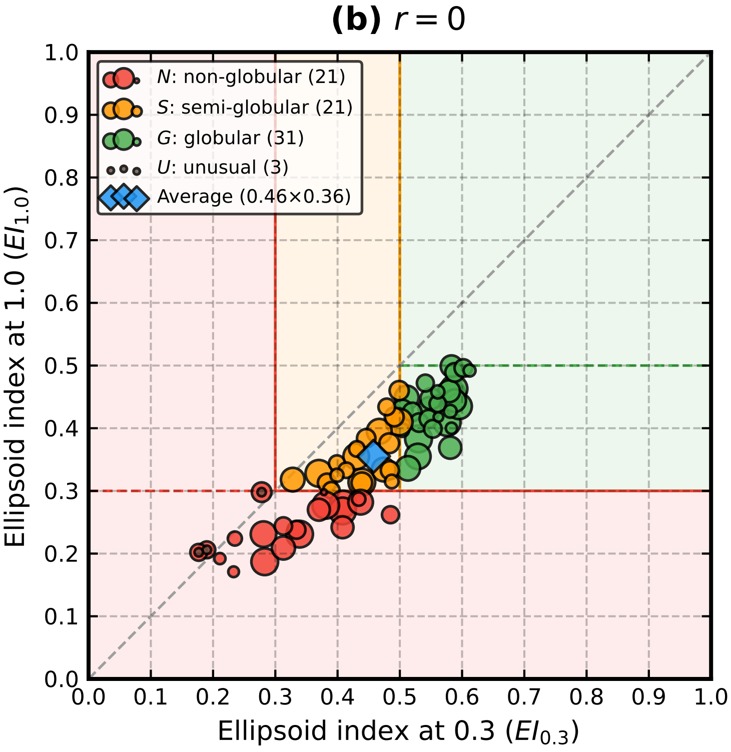

Figure S24. SCOP class f – membrane and cell surface proteins

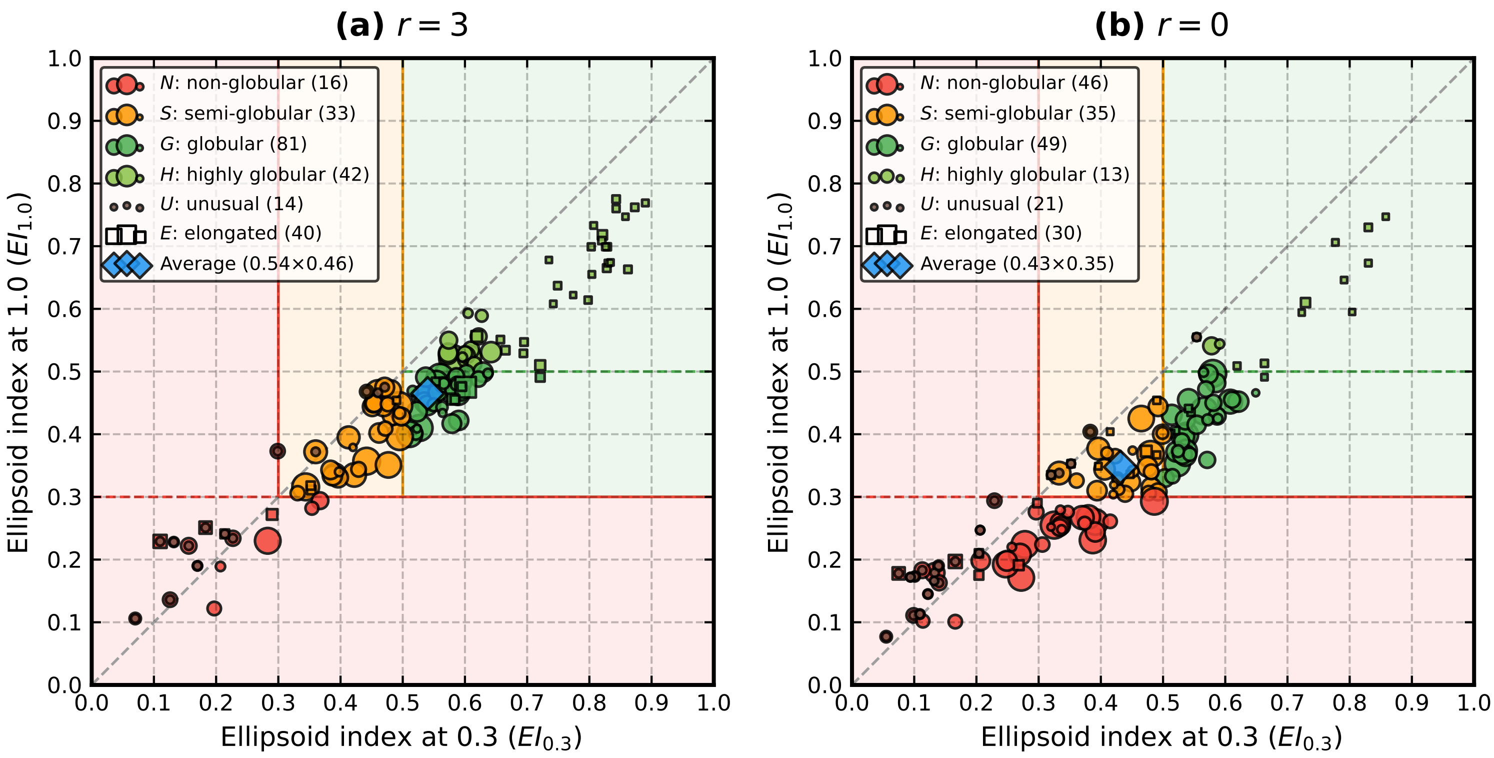

Figure S25. SCOP class g – small proteins

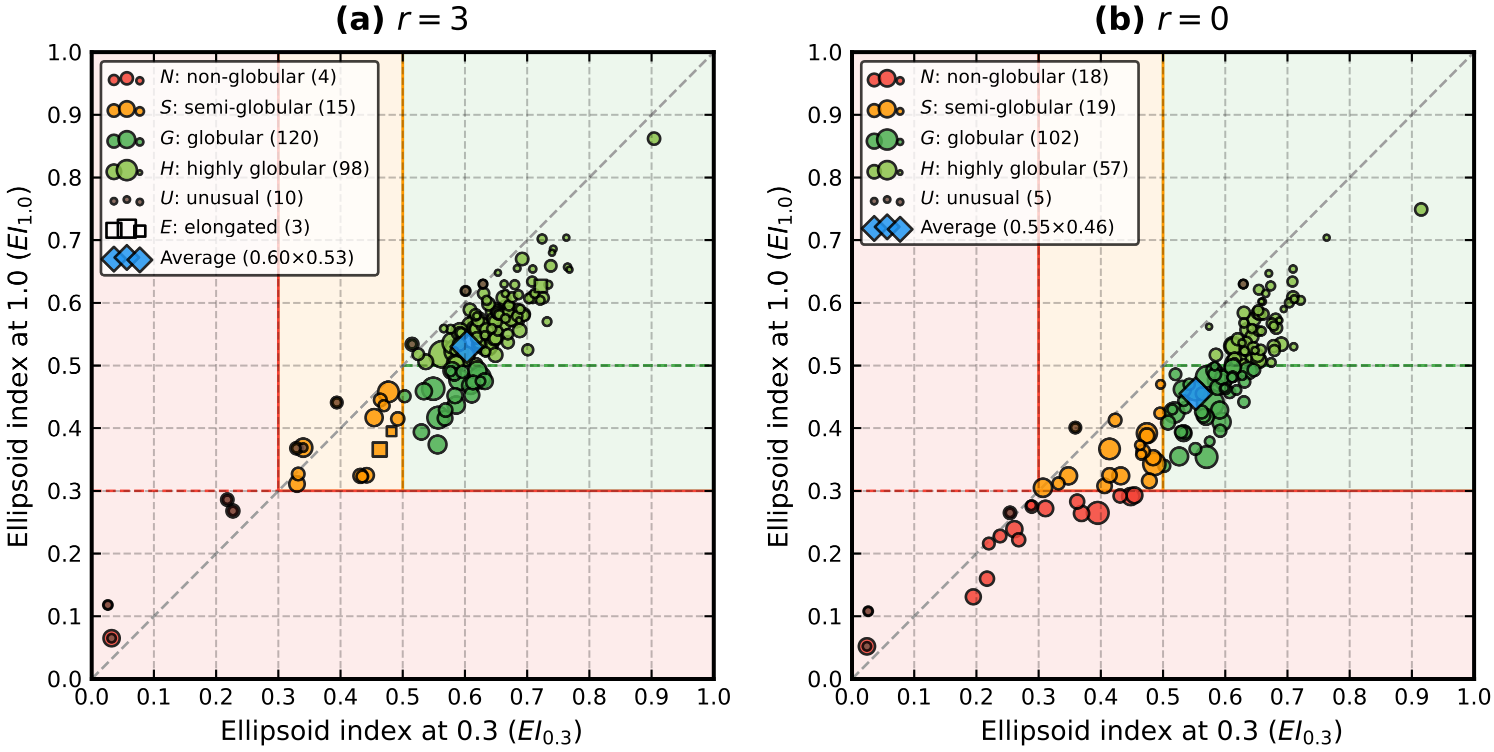

Figure S26. SCOP class h – coiled coil proteins

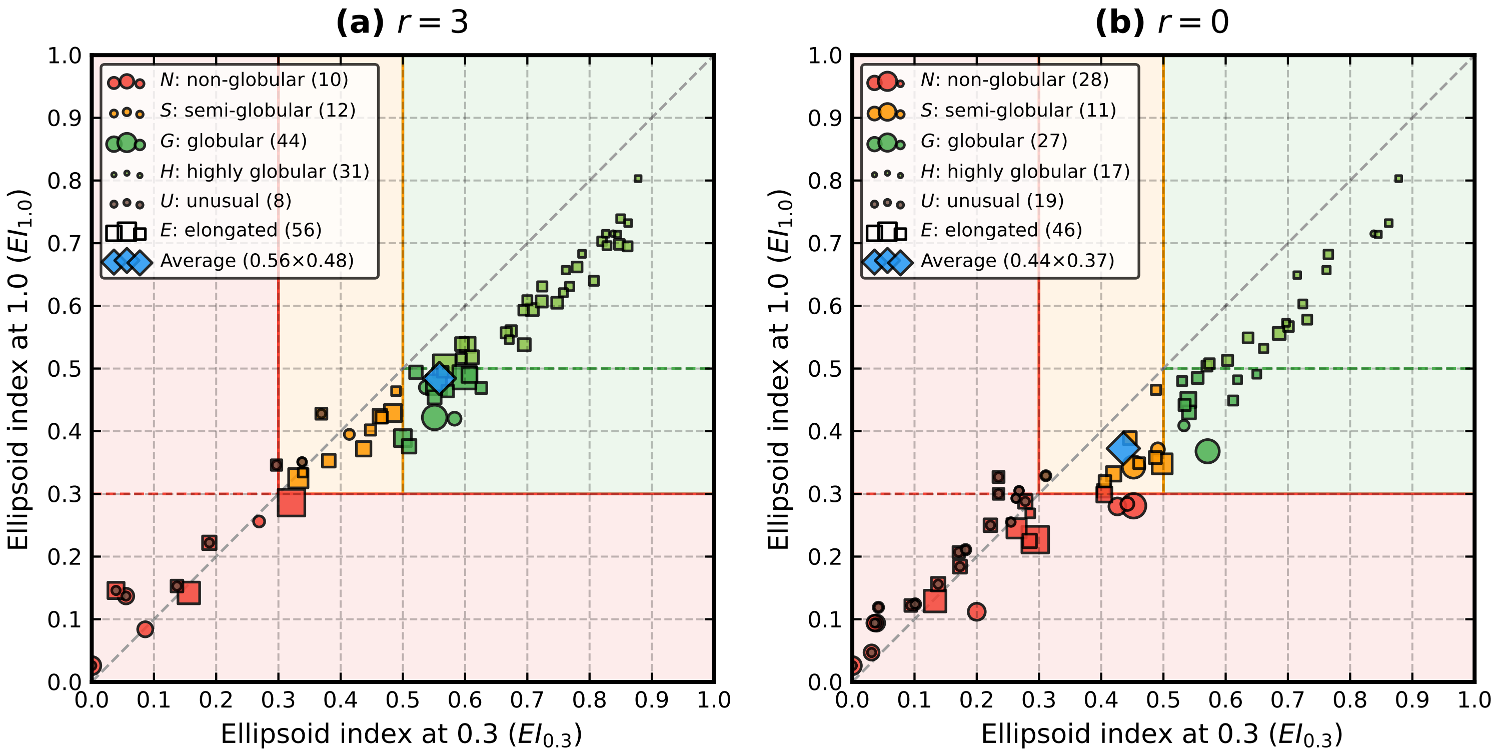

Supplement: Supplementary file 1 [file biomolecules-13-00385-s001.zip › s4.pdf]
