# Supplementary material for: Improved Assessment of Globularity of Protein Structures and the Ellipsoid Profile of the Biological Assemblies from the PDB
Source: Biomolecules. 2023 Feb 17;13(2):385. doi: 10.3390/biom13020385 (PMC9953691; doi:10.3390/biom13020385)

Figure S27. Asymmetric, 1 chain, 1 domain (A1o)

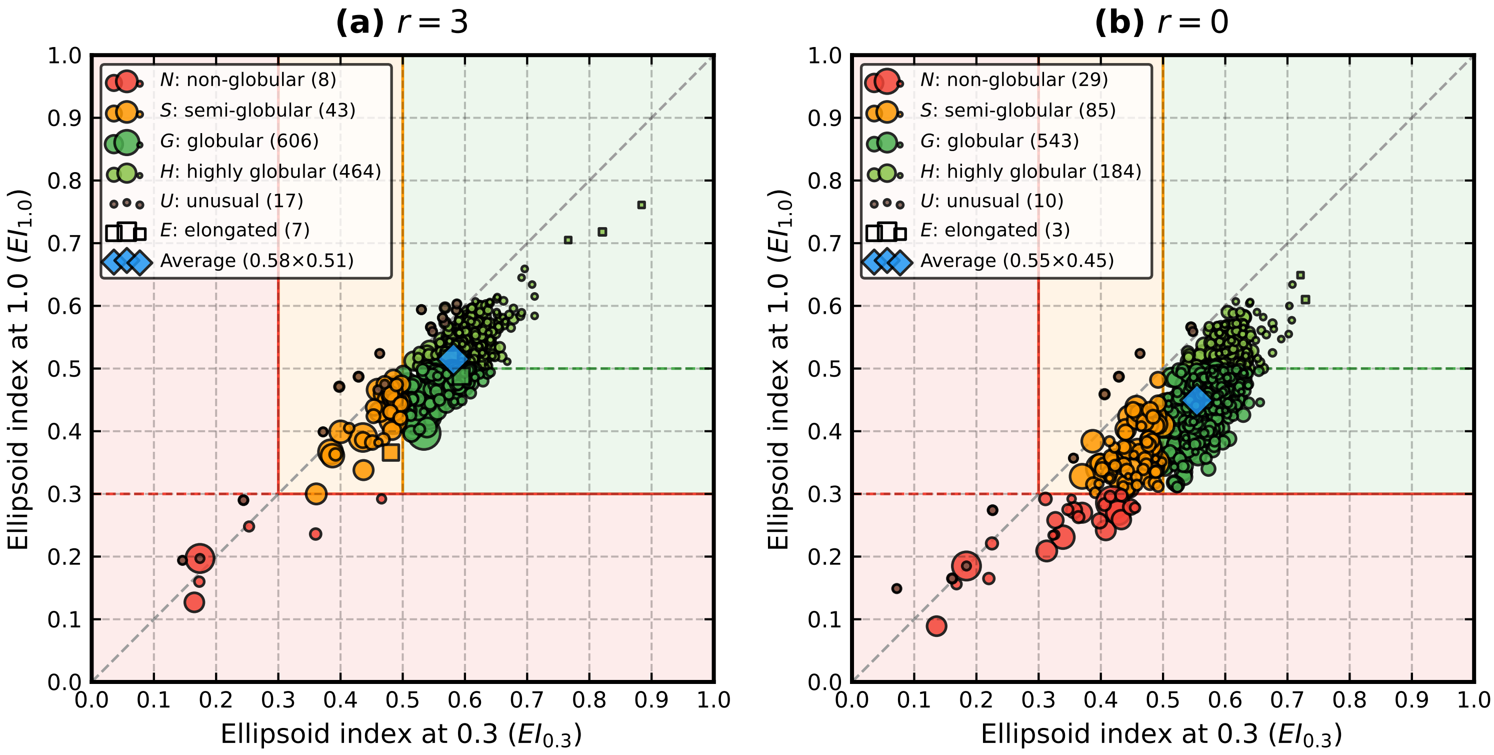

Figure S28. Asymmetric, 1 chain, multiple domains (A1m)

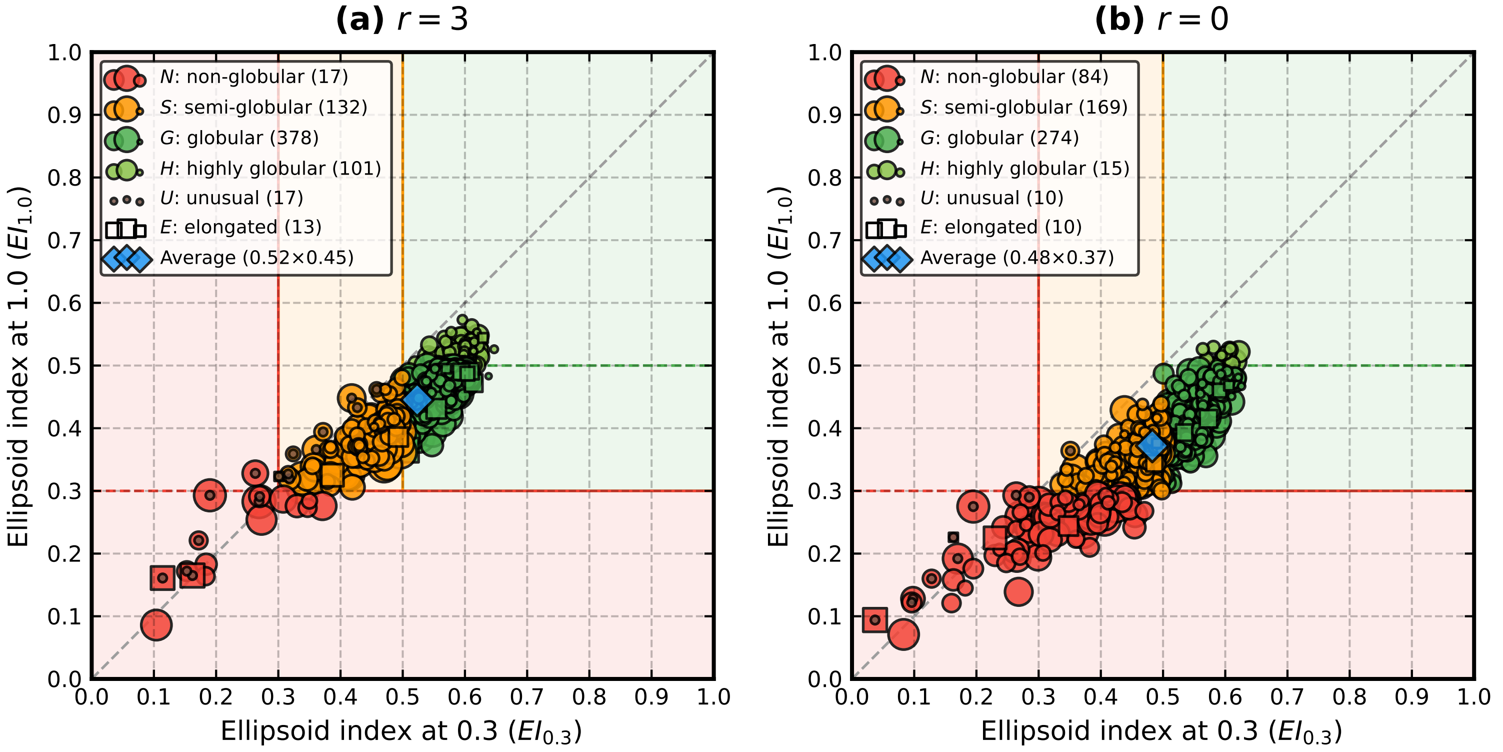

Figure S29. Asymmetric, 2 chains, 1 or more domains per chain (A2)

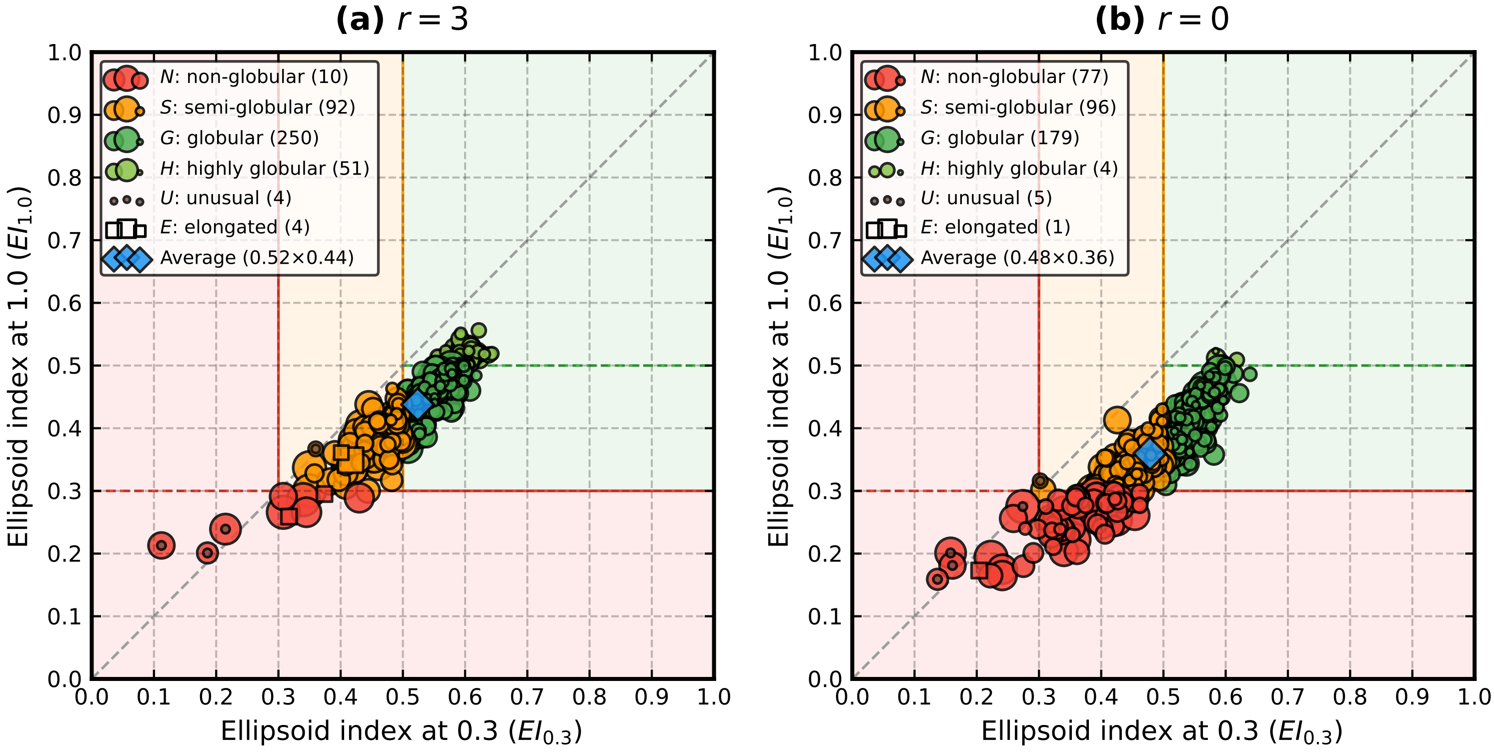

Figure S30. Asymmetric, 3 chains, 1 or more domains per chain (A3)

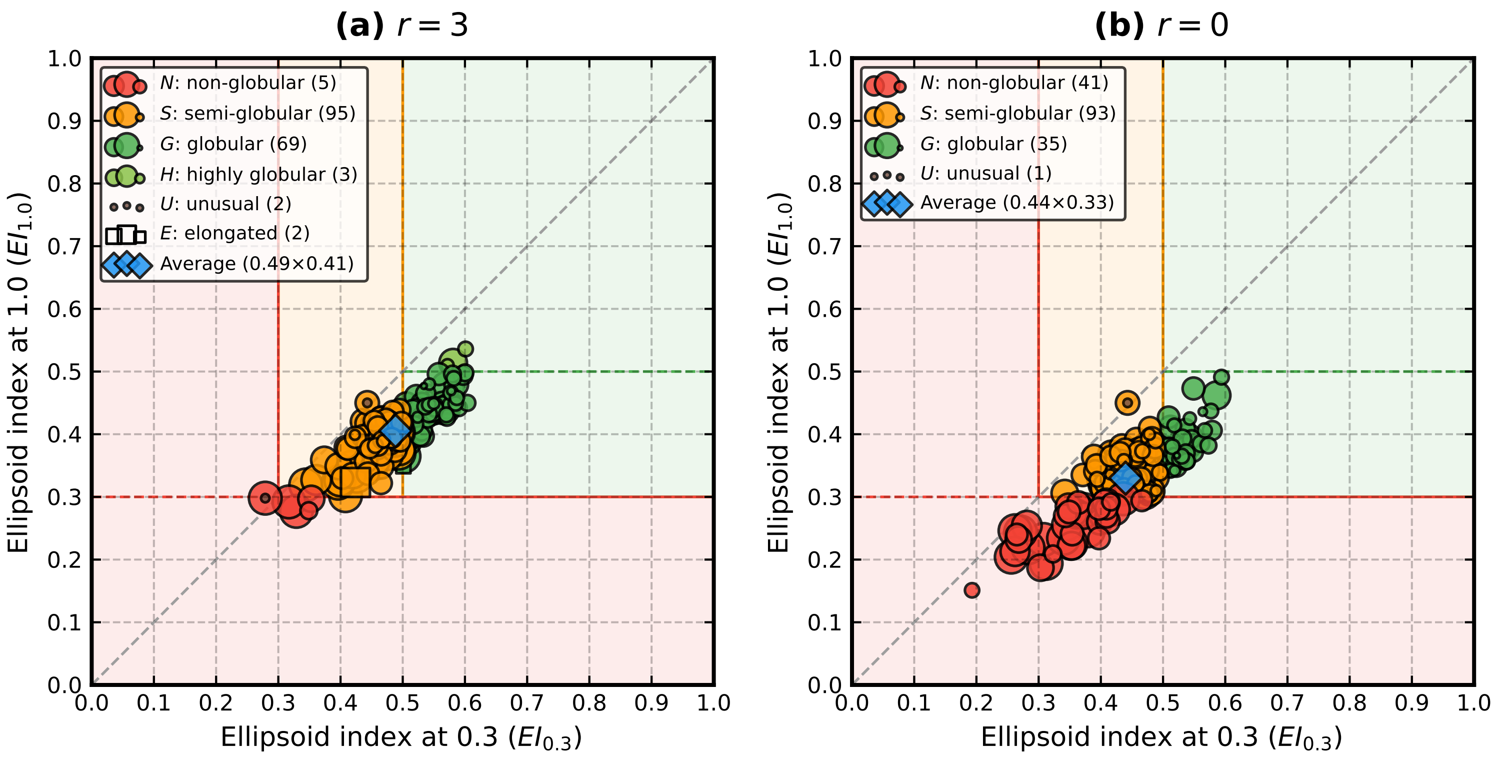

Figure S31. Asymmetric, 4 or more chains, 1 or more domains per chain (A4+)

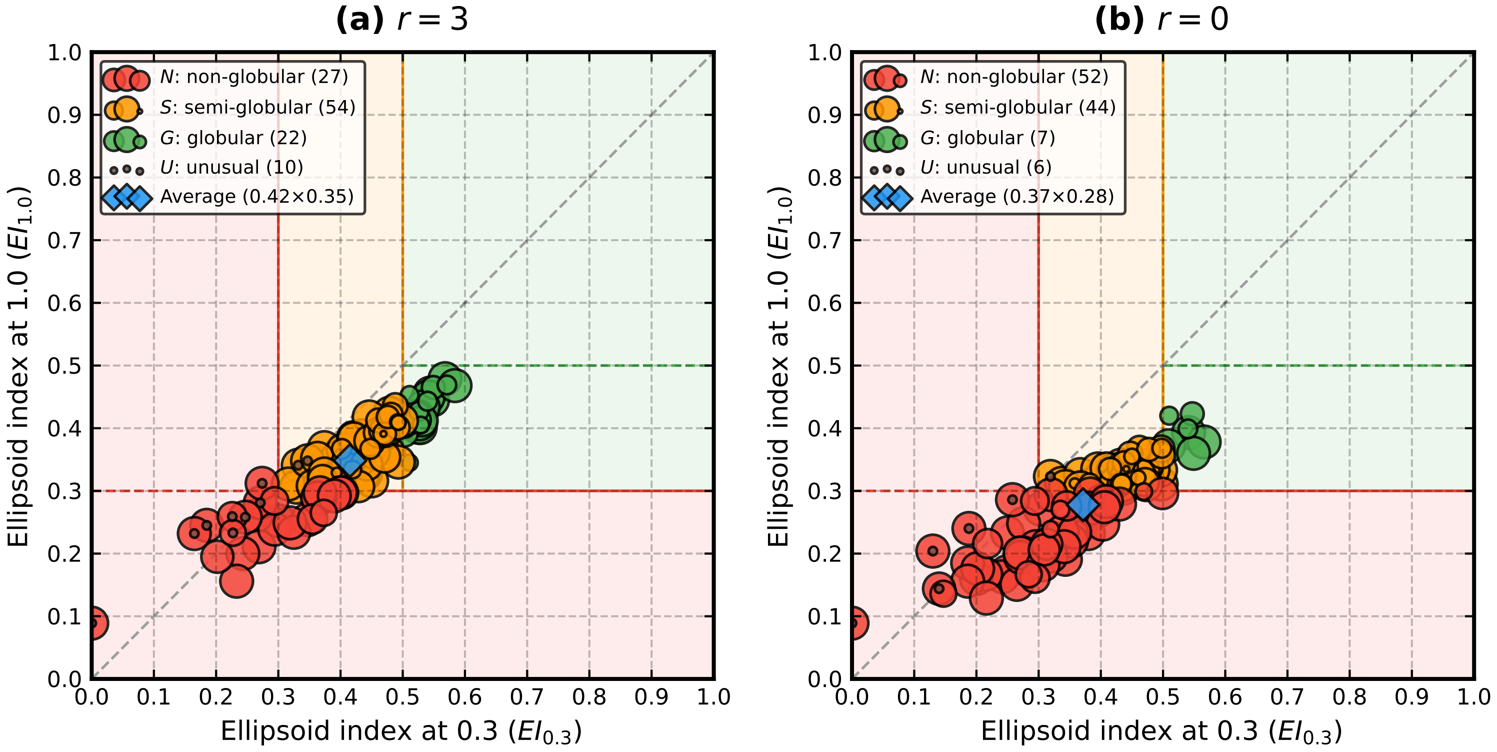

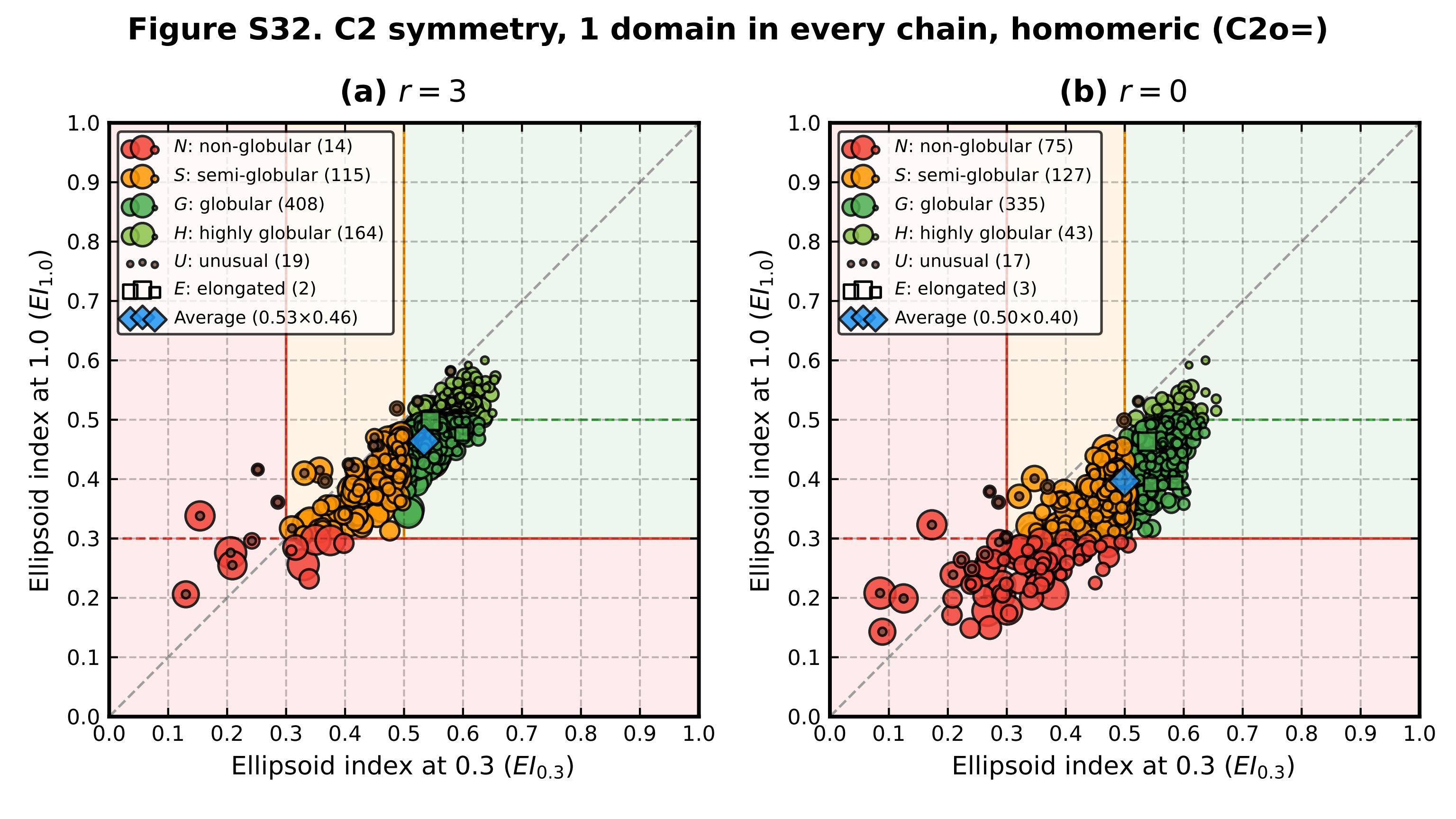
Figure S32. C2 symmetry, 1 domain in every chain, homomeric (C2o=)
(a) r = 3
(b) r = 0
N: non-globular (14)
S: semi-globular (115)
G: globular (408)
H: highly globular (164)
U: unusual (19)
E: elongated (2)
Average (0.53×0.46)
N: non-globular (75)
S: semi-globular (127)
G: globular (335)
H: highly globular (43)
U: unusual (17)
E: elongated (3)
Average (0.50×0.40)
Ellipsoid index at 1.0 (EI1.0)
Ellipsoid index at 0.3 (EI0.3)
0.0
0.1
0.2
0.3
0.4
0.5
0.6
0.7
0.8
0.9
1.0

Figure S33. C2 symmetry, multiple domains in some chains, homomeric (C2m=)

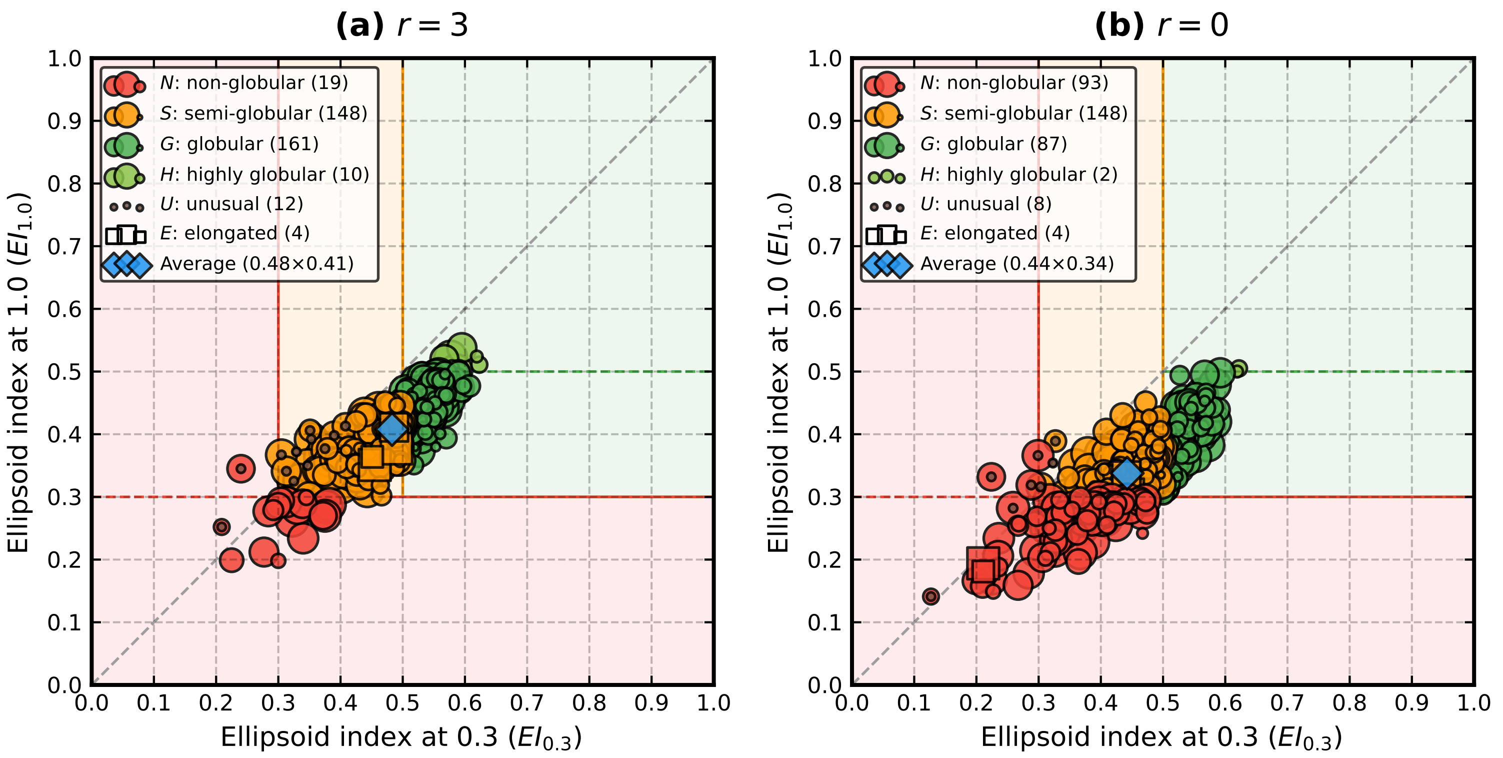

Figure S34. C2 symmetry, 1 domain in every chain, heteromeric (C2o≠)

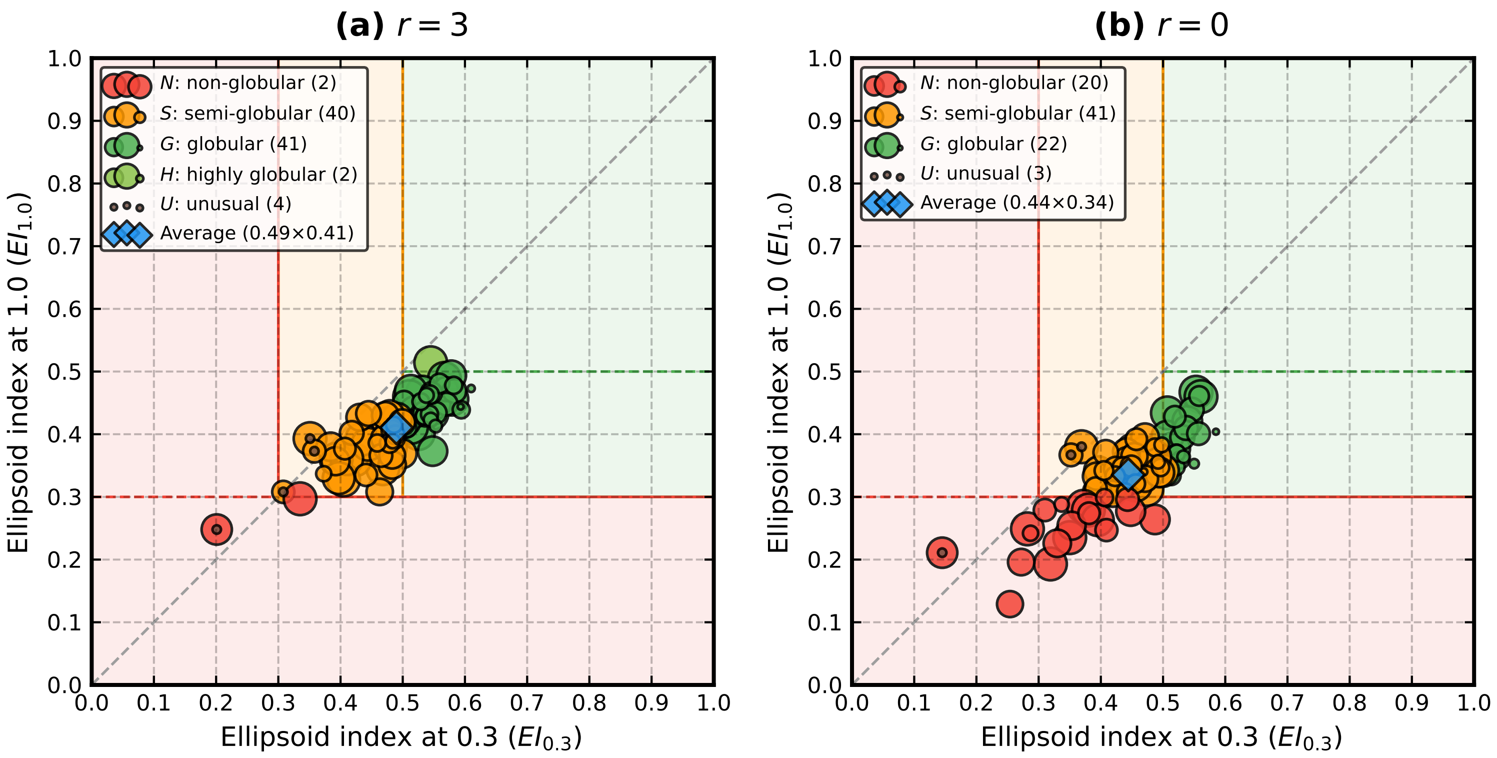

Figure S35. C2 symmetry, multiple domains in some chains, heteromeric (C2m≠)

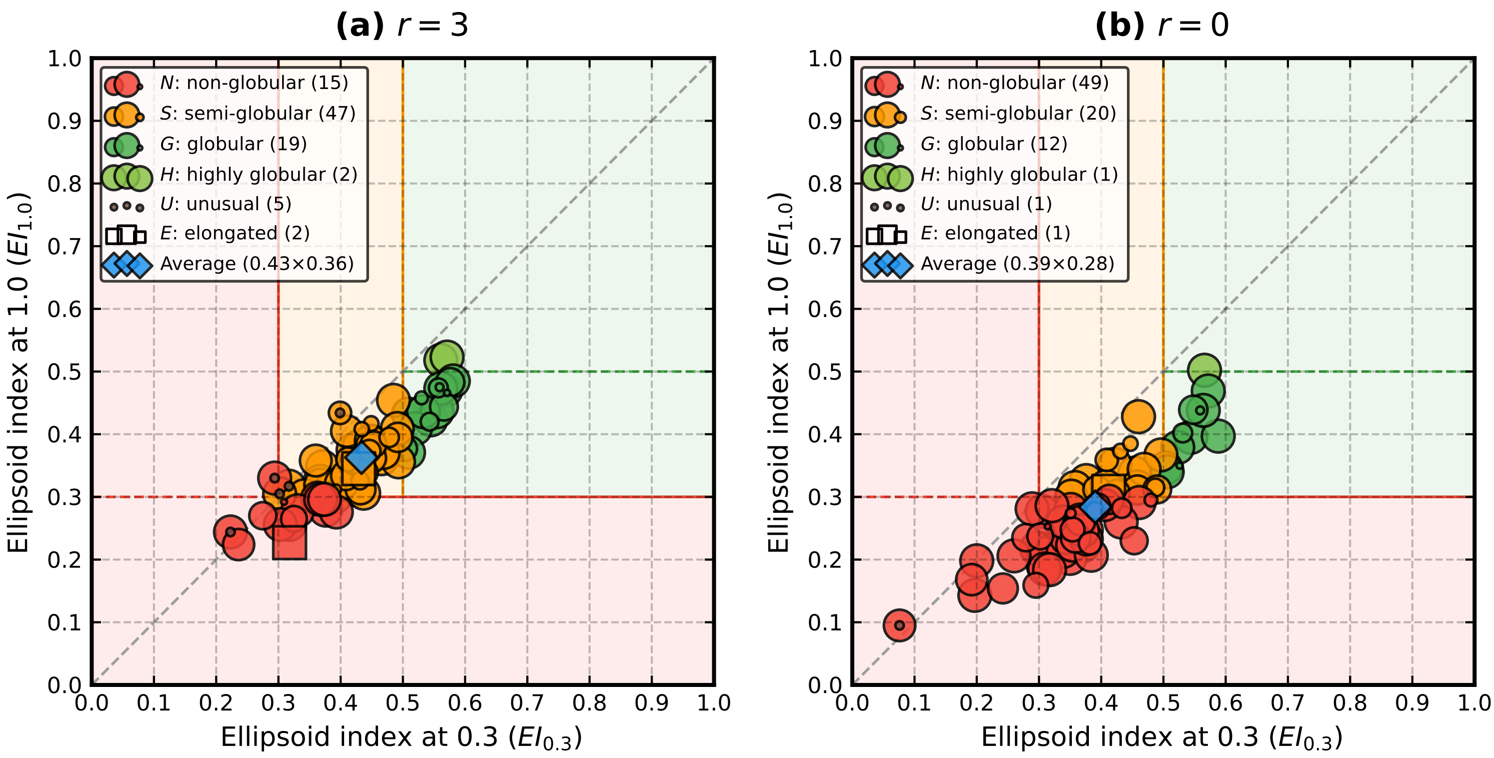

Figure S36. C3 symmetry, 1 or more domains per chain (C3)

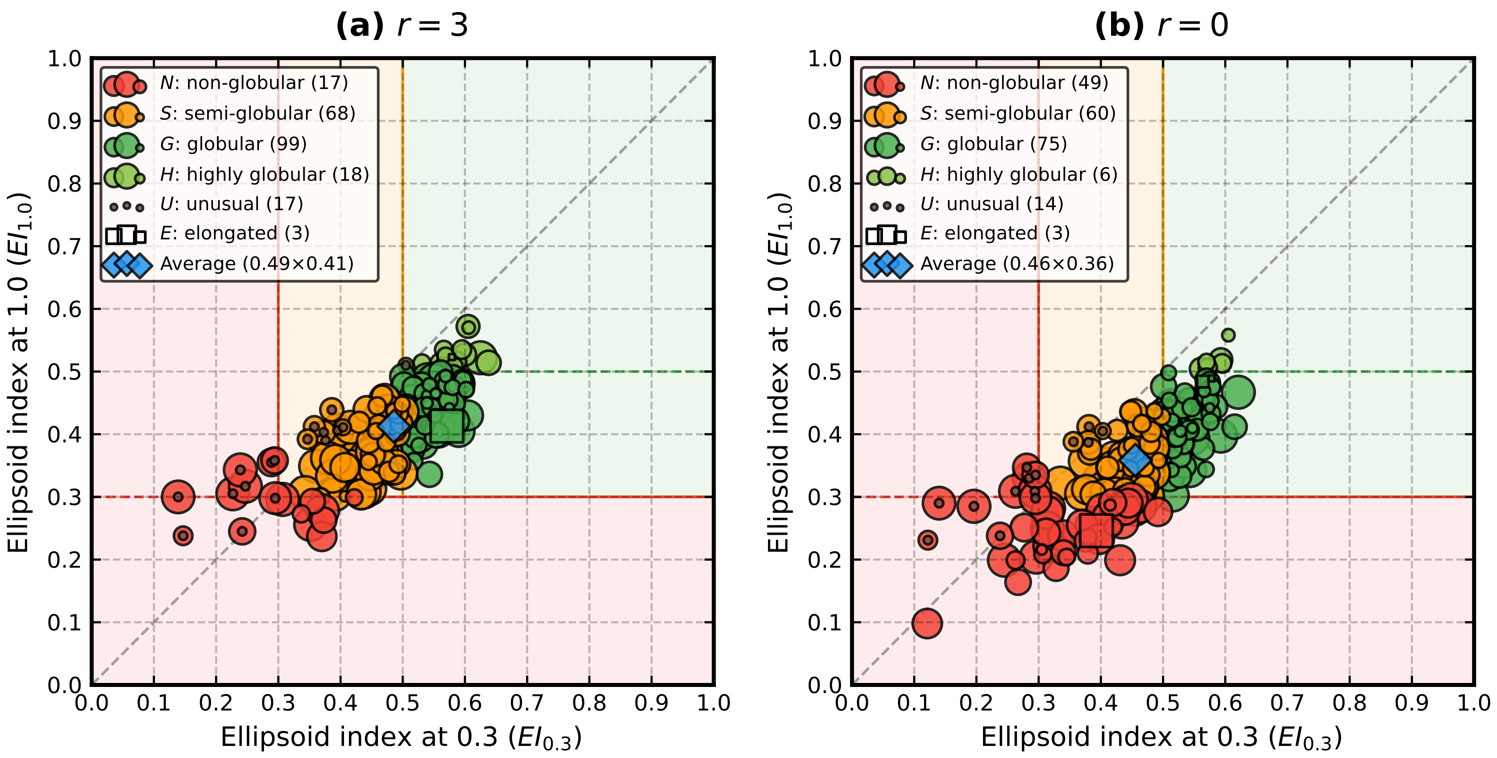

Figure S37. C4 or higher symmetry, 1 or more domains per chain (C4+)

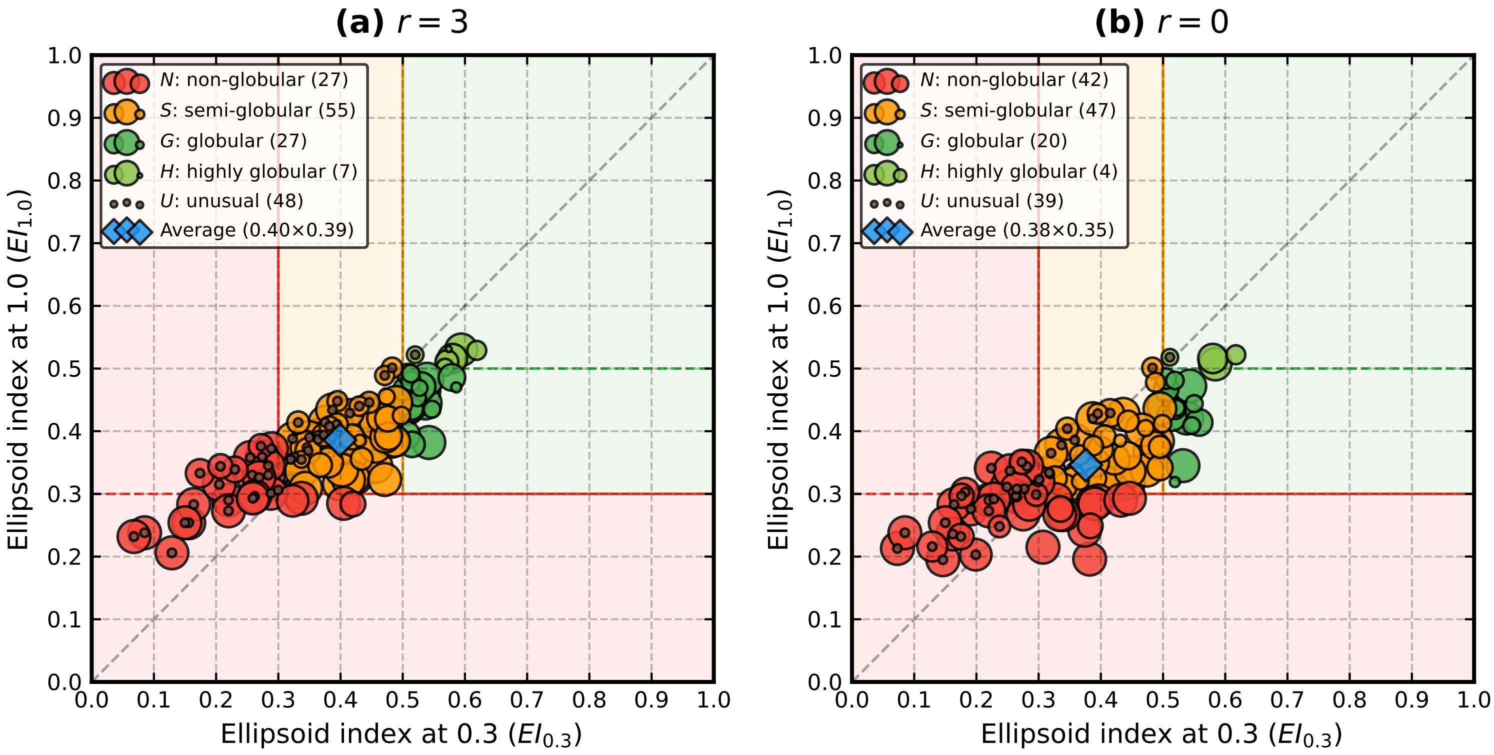

Figure S38. D2 symmetry, 1 or more domains per chain (D2)

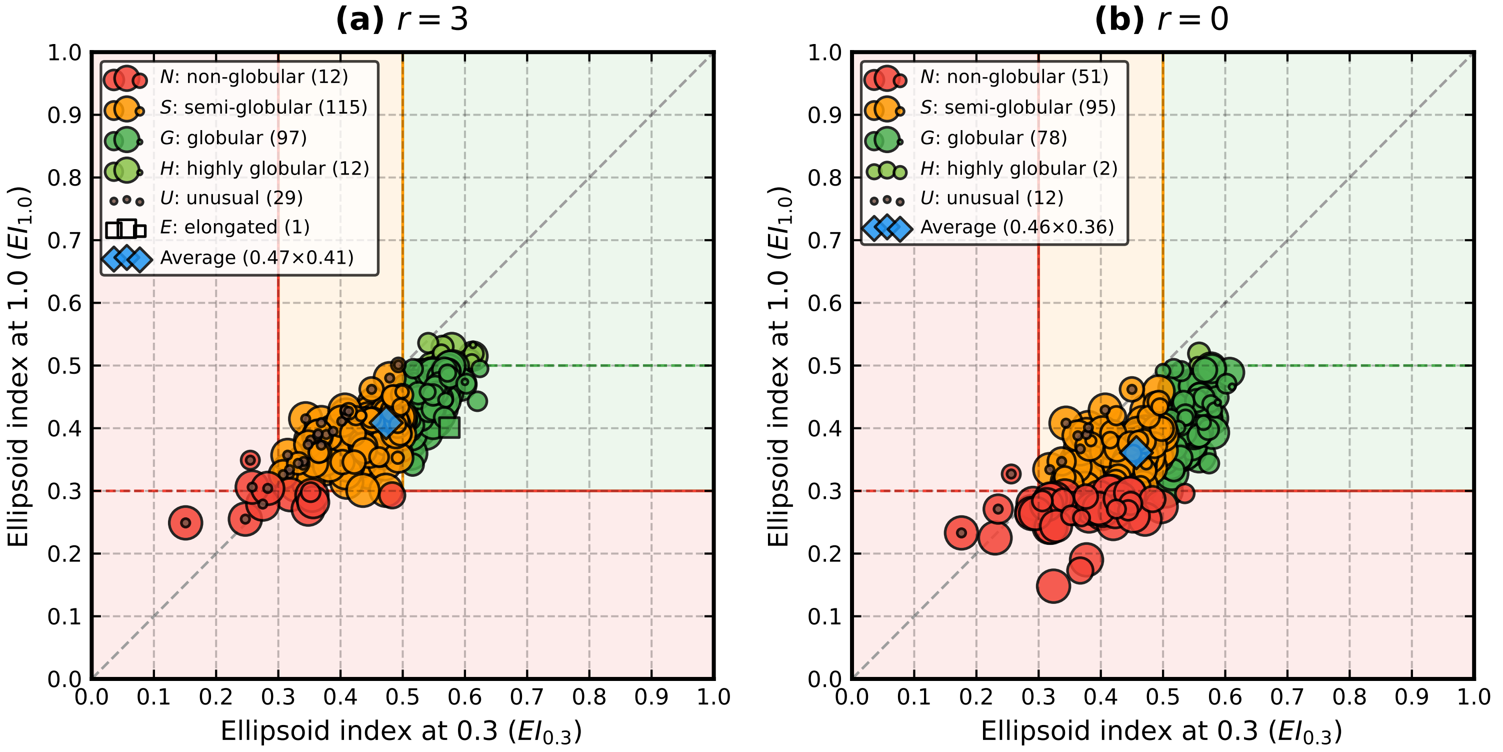

Figure S39. D3 symmetry, 1 or more domains per chain (D3)

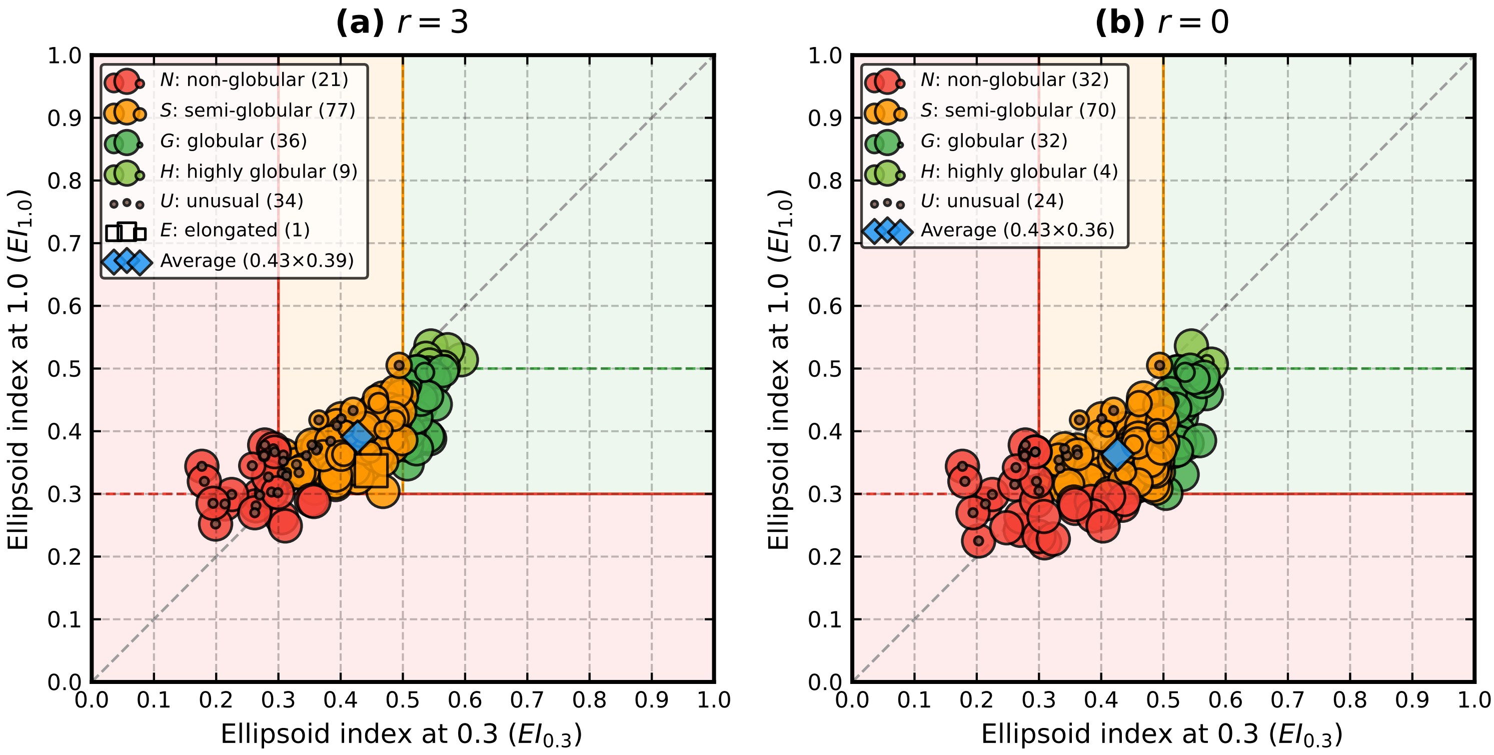

Figure S40. D4 or higher symmetry, 1 or more domains per chain (D4+)

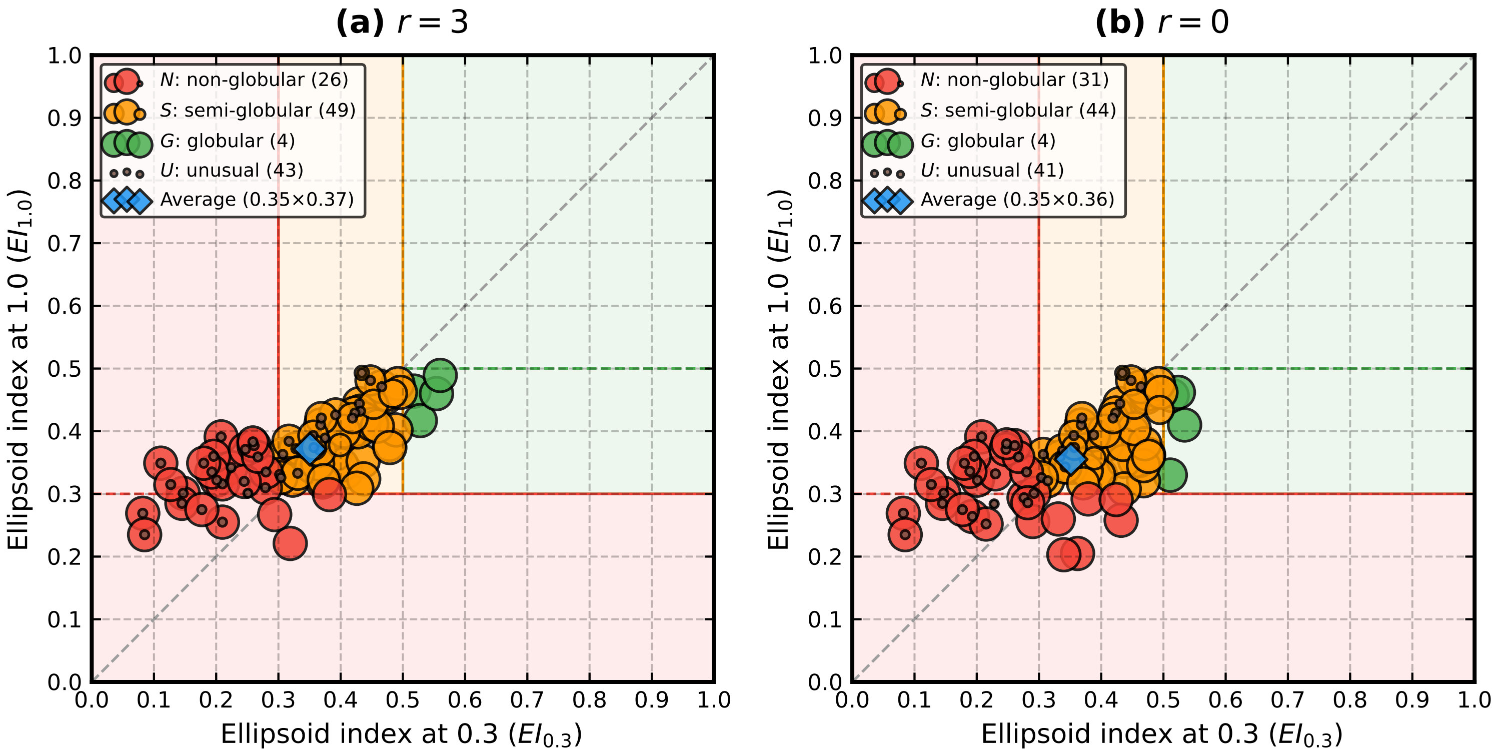

Figure S41. Tetrahedral or octahedral or icosahedral symmetry (TOI)

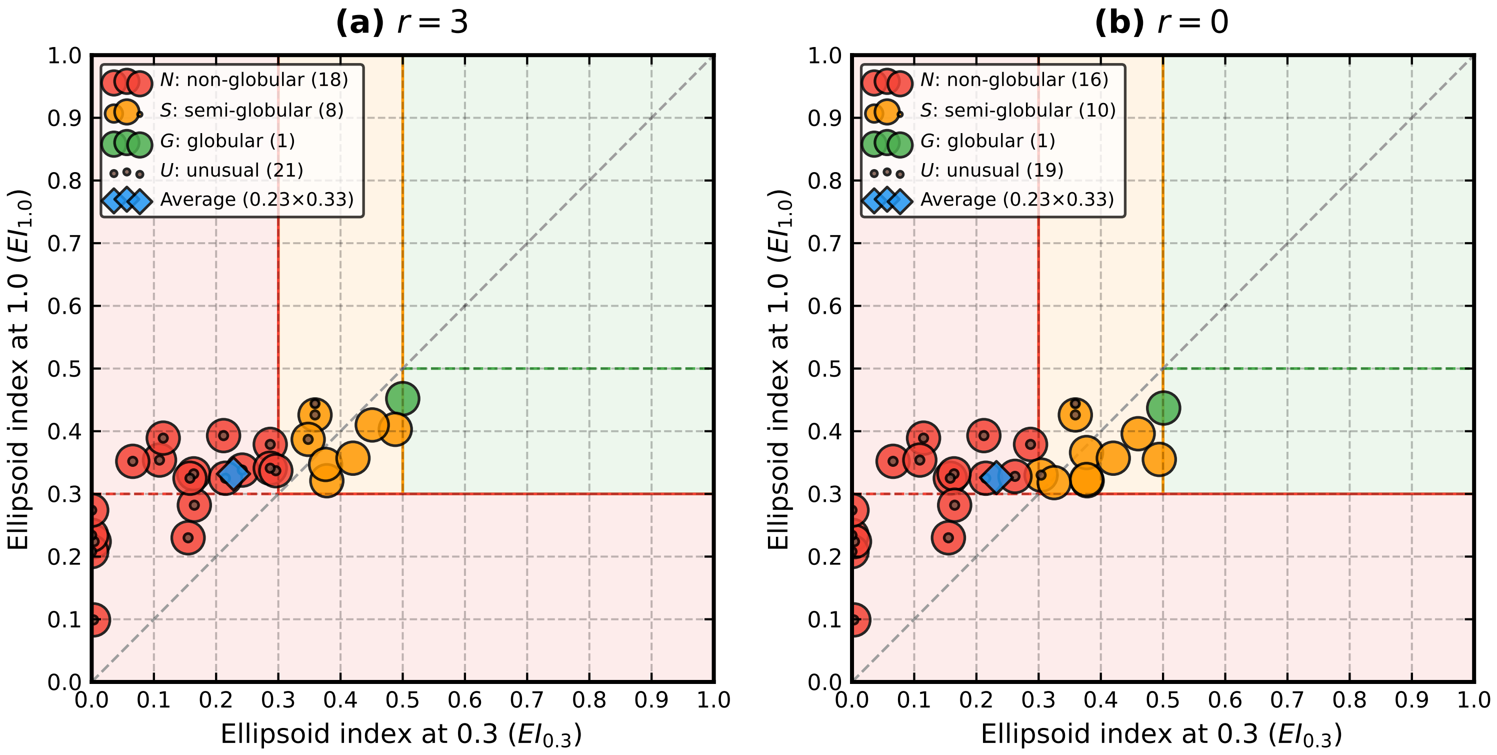

Supplement: Supplementary file 1 [file biomolecules-13-00385-s001.zip › s7.pdf]
